# Association between cheese and fish consumption and the occurrence of depression based on European population: mediating role of metabolites

**DOI:** 10.3389/fnut.2024.1322254

**Published:** 2024-04-17

**Authors:** Yan Chen, Jixin Lin, Ming Tao

**Affiliations:** ^1^Second Clinical Medical School, Zhejiang Chinese Medical University, Hangzhou, China; ^2^Second Affiliated Hospital, Zhejiang Chinese Medical University, Hangzhou, China

**Keywords:** cheese, fish, mediation, Mendelian randomization, metabolites, depression

## Abstract

**Background:**

The consumption of cheese and fish has been linked to the onset of depression. However, the connection between consuming cheese, consuming fish, experiencing depression, and the pathways that mediate this relationship remains unclear. The purpose of this research was to investigate the potential association between the consumption of cheese and fish and the occurrence of depression. Moreover, it is important to identify any metabolites that might be involved and understand their respective roles and functions.

**Methods:**

A two-step, two-sample Mendelian randomization (MR) study was conducted using genome-wide association study (GWAS) data on cheese, non-oily fish, and oily fish consumption and depression, along with 12 alternate mediators. The study included a total of 451,486 participants in the cheese consumption group, 460,880 in the non-oily fish consumption group, 460,443 in the oily fish consumption group, and 322,580 with a diagnosis of depression. The single nucleotide polymorphism (SNP) estimates were pooled using inverse-variance weighted, weighted median, MR-Egger, simple mode, and weighted mode.

**Results:**

The data we collected suggested that consuming more cheese correlated with a lower likelihood of experiencing depression (OR: 0.95; 95% CI: 0.92 to 0.98). Neither non-oily fish nor oily fish consumption was directly linked to depression onset (*p* = 0.08, *p* = 0.78, respectively). Although there was a direct causal relationship with depression, the mediating relationship of triglycerides (TG), total cholesterol in large HDL, cholesterol to total lipids ratio in large HDL, free cholesterol to total lipids ratio in large HDL, glycine, and phospholipids to total lipids ratio in very large HDL of cheese intake on depression risk were − 0.002 (95% CI: −0.023 - 0.020), −0.002 (95% CI: −0.049 - 0.045), −0.001 (95% CI: −0.033 - 0.031), −0.001 (95% CI: −0.018 - 0.015), 0.001 (95% CI: −0.035 - 0.037), and − 0.001 (95% CI: −0.024 - 0.021), respectively. The mediating relationship of uridine, free cholesterol to total lipids ratio in large HDL, total cholesterol in large HDL, acetoacetate, and 3-hydroxybutyrate (3-HB) between non-oily fish consumption and depression risk were 0.016 (95% CI: −0.008 - 0.040), 0.011 (95% CI: −1.269 - 1.290), 0.010 (95% CI: −1.316 - 1.335), 0.011 (95% CI: −0.089 - 0.110), and 0.008 (95% CI: −0.051 - 0.068), respectively. The mediation effect of uridine and free cholesterol to total lipids ratio in large HDL between intake of oily fish and the risk of depression was found to be 0.006 (95% CI: −0.015 - 0.028) and − 0.002 (95% CI: −0.020 - 0.017), respectively. The correlation between eating cheese and experiencing depression persisted even when adjusting for other variables like Indian snacks, mango consumption, sushi consumption, and unsalted peanuts using multivariable MR.

**Conclusion:**

The consumption of cheese and fish influenced the likelihood of experiencing depression, and this may be mediated by certain metabolites in the body. Our study provided a new perspective on the clinical treatment of depression.

## 1 Introduction

Depression, which is linked to an increased risk of suicide, is a leading problem for global public health (1). Affecting approximately 350 million people worldwide, depression is a widely acknowledged and complex mental health issue (2–4). It is expected that this number will increase, especially due to the COVID-19 pandemic and its consequences, which have notably influenced the younger population and caused a spike in the number of cases among this group (5). In China, the prevalence of depression poses a significant concern and a notable challenge. Research findings specifically reveal that as many as 23.6% of adults exhibit symptoms associated with depression, a percentage that not only increases with age but also shows a worrying trend in young people. A meta-analysis focusing on Chinese adolescents revealed a depression prevalence rate of 24.3% (6–9). Understanding the possible causes and consequences of depression may be helpful for implementing prevention strategies. There has been much research on the causes of depression, but only stressors, certain sociodemographic factors, and family history are clearly indicated (10). Nutritional factors significantly contribute to mental health problems, while others help in maintaining good mental health. During the past decades, researchers have paid much attention to the link between dietary intake and depression.

Dairy products contain a high amount of important nutrients, such as vitamins B2 and B12, magnesium, potassium, phosphorus, protein, zinc, and calcium (11). The crucial role of calcium in preserving the health of the nervous system cannot be overstated. A lack of calcium has been shown to play a significant role in the onset of neurological disorders, including depression (12). This connection highlights the importance of dairy products in a balanced diet for maintaining mental well-being. Dairy products mainly include milk, cheese, ice cream, and yogurt. Research on cheese intake is relatively extensive. The relationship between cheese consumption and depression remains controversial. A meta-analysis involving eight studies and a total of 83,533 participants suggested that those who consumed cheese had a lower risk of depression. After conducting a comprehensive analysis, it was confirmed that there was a notable correlation between cheese consumption and depression [odds ratio (OR) = 0.91, 95% confidence interval (CI) = 0.84–0.98] (13). However, additional research findings suggest a potential connection between increased cheese consumption and an increase in stress levels. For instance, in a cross-sectional study of 7,387 adults, researchers discovered a correlation between high cheese consumption and elevated levels of stress (OR = 1.52, 95% CI = 1.02–2.26) (14). Moreover, another study showed no significant relationship between cheese consumption and depression. Cheese intake was assessed by providing complete data on a 24-h dietary recall questionnaire, sociodemographic information, and lifestyle factors, and by completing the Patient Health Questionnaire (PHQ-9) for depression screening (15). Previous studies have been based on clinical or animal experiments, which inevitably introduce systematic errors and confounding factors, leading to varying, and sometimes controversial, research results.

Mendelian randomization (MR) is based on the random allocation of single nucleotide polymorphisms (SNPs) during the formation of gametes to form zygotes. It eliminates confounding factors, thus enhancing the reliability and comparability of findings. Fish consumption can be divided into non-oily and oily fish consumption. Fish, particularly salmon and snapper, are a key source of omega-3 fatty acids that promote heart health by converting alpha-linolenic acid (ALA) into long-chain omega-3 fatty acids. A lack of omega-3 fatty acids, specifically eicosapentaenoic acid (EPA) and docosahexaenoic acid (DHA), has been linked to impaired neuronal function and alterations in inflammatory reactions (16). Although research substantiates the preventive and therapeutic benefits of omega-3 polyunsaturated fatty acids (PUFAs) in addressing depression, the exact mechanisms remain incompletely understood but are believed to be related to physiological pathways that involve fatty acids (17). A previous study of 15,000 adults revealed that there was no substantial correlation between fish consumption and depression (18). However, results from the MEDiterranean ISlands (MEDIS) Elderly Epidemiological Study showed that continual consumption of fish was related to less severe symptoms of depression in elderly individuals (19). These results underscore the inconsistent findings in research regarding the relationship between fish consumption and depression.

Targeted metabolomics and peptidomics have emerged as powerful methodologies for gaining a comprehensive understanding of the intricate biochemical alterations within food systems. Cheese and fish consumption can indeed lead to changes in metabolites within the body, which can subsequently affect various metabolic pathways (20, 21). Different metabolites belong to the corresponding metabolic pathways. Moreover, metabolic pathways are usually regulated by enzymes. The synthesis and degradation of ketone bodies provide a vital energy source. Butanoate is involved in the metabolism of fatty acids and glucose. Amino acid metabolism is involved in various biosynthetic pathways. Pyrimidine metabolism plays a pivotal role in nucleic acid synthesis and cellular proliferation. Steroid biosynthesis is crucial for maintaining physiological equilibrium. Changes in metabolites and the disruption of metabolic pathways have been shown to be correlated with the pathogenesis of depression (22).

Due to the expense and time involved with most randomized controlled studies, substitution methods to reinforce a causal relationship could aid in determining whether such trials are worthwhile. Genetic variants are used as instrumental variables (IVs) in MR to determine causal relationships between exposures and outcomes (23). During meiosis and fertilization, genetic variants are assigned randomly and established well before the disease develops. Therefore, they are relatively independent of self-selected behaviors and eliminate the problem of confounding factors and reverse causality (24). Two-sample Mendelian randomization analysis can be performed using the genome-wide association study (GWAS) data, which has recently been used for cheese and fish consumption and depression in our study, and we focused on metabolites as mediators and investigated the metabolic pathways associated with beneficial metabolites. We examined the causality of cheese and fish consumption on depression risk and investigated the mediating pathways through metabolite-related phenotypes using the two-sample, two-step MR analysis.

## 2 Materials and methods

### 2.1 Study design

**Figure 1:**
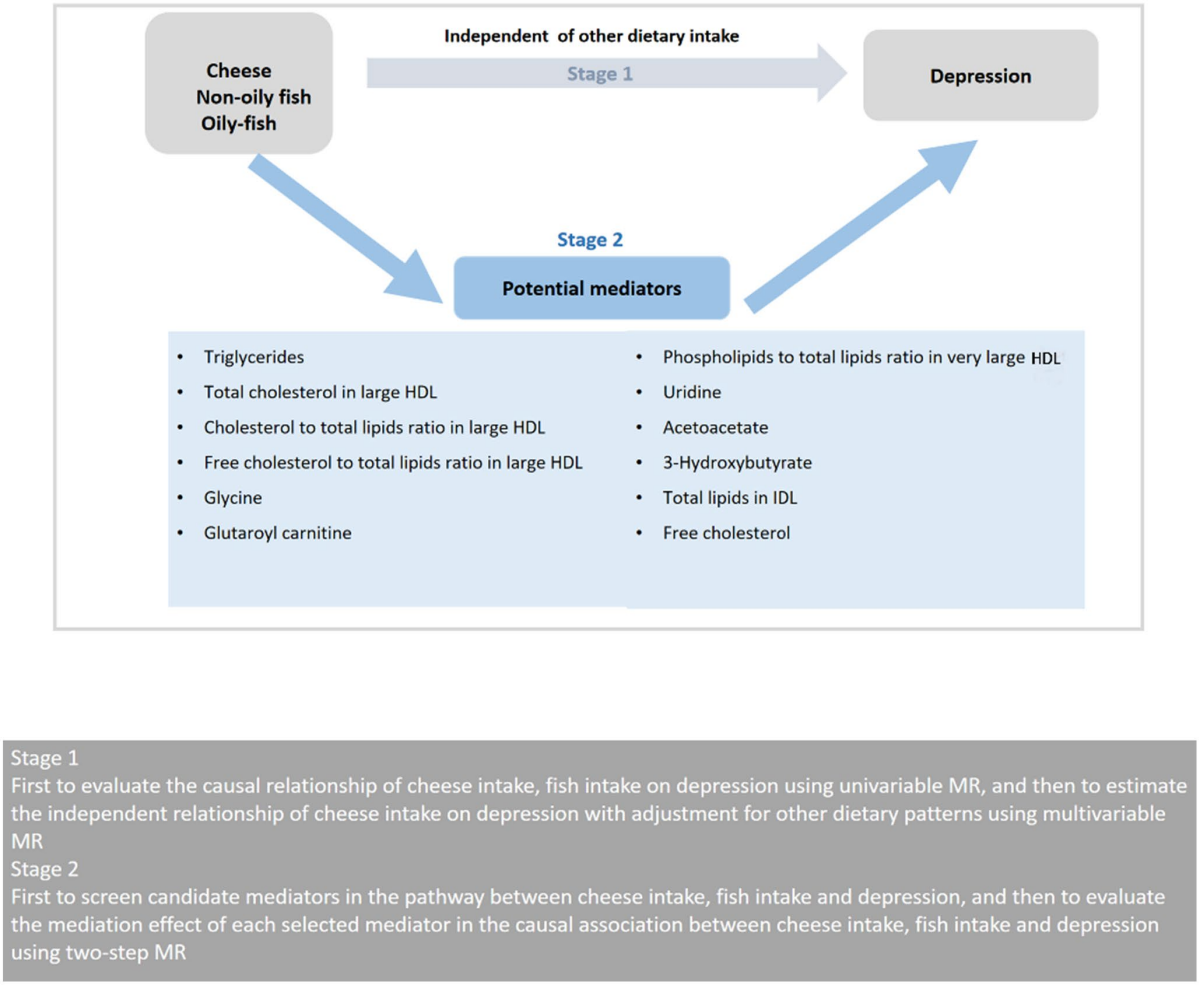
Overview of the MR study design. HDL, high-density lipoprotein; MR, Mendelian randomization.

The study was conducted in two stages as illustrated in Figure 1. In the first stage, we conducted a two-sample univariable MR utilizing the GWAS summary statistics to examine the possible causal link between cheese and fish consumption and depression. We then used multivariable MR to evaluate the effect of cheese consumption on depression with adjustment for other dietary intake, including Indian snacks, mango, sushi, and unsalted peanuts. In the second stage, we chose 12 possible mediators associated with diet and depression, involving 9 mediators for cheese consumption, 5 mediators for non-oily fish consumption, and 2 mediators for oily fish consumption. We then used a two-step MR to determine the mediating roles of selected mediators on the causal link between cheese and fish consumption and depression.

The three fundamental assumptions of MR were strictly followed in this study. First, the instrumental variables (IVs) had a strong correlation with exposure. Second, the IVs remained unaffected by any potential confounding variables that could impact the relationship between the exposure and the final result. Third, the IVs only influenced the outcome via exposure. Summary data from GWASs were used in this MR study, which was based primarily on studies of Europeans or consortia of Europeans.

### 2.2 Data sources

This analysis mainly utilized the GWAS statistical data for European populations, including individuals of both genders. The United Kingdom Biobank study provided summary statistics on cheese consumption, with a sample size of 451,486 individuals. It examined the link between cheese consumption and SNPs (25). Data are already available from a GWAS (26). Non-oily fish consumption (*n* = 460,880)^1^ and oily fish consumption (*n* = 460,443)^2^ were also obtained from the United Kingdom Biobank study. Six lipid traits were studied from the GWAS (78,700 individuals were involved in studying TG within the family GWAS consortium, 21,558 individuals for total cholesterol in large HDL (27), 115,078 individuals for cholesterol to total lipids ratio in large HDL, 115,078 individuals for free cholesterol to total lipids ratio in large HDL, 115,053 individuals for phospholipids to total lipids ratio in very large HDL and 113,595 individuals for 3-HB), as well as glycine and acetoacetate, which were studied in 114,972 and 115,075 individuals, respectively. Additionally, uridine was investigated using data from a GWAS database of approximately 7,800 individuals (28). SNPs associated with depression were extracted from a GWAS of 113,769 depression cases and 208,811 controls of European ancestry. We imported the IVs into the PhenoScanner V2, and SNPs that were associated with depression were eliminated. Indian snacks (*n* = 64,949) and intake of mango (*n* = 64,949), sushi (*n* = 64,949), and unsalted peanuts (*n* = 64,949) were selected. The data came from the MRC-IEU database and were mainly for the European population, including both men and women. The number of SNPs on each instrumental variable was 9,851,867. Ethical approval was not necessary for the current analysis as all the included GWAS data were publicly available and had been approved by the relevant ethical review boards.

### 2.3 Selection and validation of SNPs

Table 1 presents the data sources for each of the phenotypes investigated. The respective GWAS datasets were utilized to identify SNPs associated with cheese and fish consumption, and each mediator under consideration, using a genome-wide significance threshold of 5 × 10^−8^. The SNPs were grouped into clusters based on a linkage disequilibrium threshold of *r*^2^ < 0.001 within 10,000 kb to identify independent genetic variants (Supplementary Table S1). MR analyses were conducted based on the remaining SNPs after harmonizing exposure and outcome. Prior to conducting the MR analysis, we guaranteed that the effects on both the exposure and outcome were linked to the same allele, which was crucial for aligning the genetic variants consistently across multiple datasets.

**Table 1:** GWAS data sources for the MR study.

Phenotype	PMID or GWAS ID	Sample size or number of SNPs	Ancestry
Exposure			
Cheese intake	ukb-b-1489	4,51,486	European
Mediator			
TG	ieu-b-4850	78,700	European
Total cholesterol in large HDL	27005778	21,558	European
Cholesterol to total lipids ratio in large HDL	met-d-L_HDL_C_pct	1,15,078	European
Free cholesterol to total lipids ratio in large HDL	met-d-L_HDL_FC_pct	1,15,078	European
Glycine	met-d-Gly	1,14,972	European
Phospholipids to total lipids ratio in very large HDL	met-d XL_HDL_PL_pct	1,15,053	European
Exposure			
Non-oily fish intake	ukb-b-17627	4,60,880	European
Mediator			
Uridine	24816252	7,800	European
Free cholesterol to total lipids ratio in large HDL	Met-d-L_HDL_FC_pct	1,15,078	European
Total cholesterol in large HDL	27005778	21,558	European
Acetoacetate	Met-d-Acetoacetate	1,15,075	European
3-HB	Met-d-bOHbutyrate	1,13,595	European
Exposure			
Oily fish intake	ukb-b-2209	4,60,443	European
Mediator			
Uridine	24816252	7,800	European
Free cholesterol to total lipids ratio in large HDL	Met-d-L_HDL_FC_pct	1,15,078	European
Outcome			
Depression	29662059	3,22,580	European

### 2.4 Mendelian randomization design and data analysis

#### 2.4.1 Univariable and multivariable MR analyses

For the univariable MR analyses, the main approach utilized was the inverse-variance weighted (IVW) method. We also conducted additional analyses using MR-Egger, weighted median, simple mode, and weighted mode approaches to assess the reliability of the IVW estimates under varying assumptions. The IVW method pools together the individual SNP-level estimate using a random-relationships meta-analysis to obtain a single estimation of causality (23). To investigate possible pleiotropic effects of IVs, MR-Egger regression analysis was utilized. Detecting directional horizontal pleiotropy in causal estimates through the intercept term in MR-Egger regression analysis can provide crucial insights into potential confounding factors impacting the results. The weighted median method identifies the median MR estimate as the causal estimate, ensuring consistency in the causal estimation when at least half of the weight is assigned to valid IVs (29). The clustering of SNPs based on the similarity of their causal relationships was carried out in both the simple mode and weighted mode approaches, with the estimation of causal relationships being conducted using the largest cluster (30). The heterogeneity of the IVW estimates was assessed using Cochran’s Q test, with statistical significance defined as *p* < 0.05; then, we detected the occurrence of horizontal pleiotropy by analyzing the intercept term. The primary approach used for the analysis of multivariable MR was the multivariable inverse-variance-weighted (MV-IVW) method. The principle of correcting for confounding factors was to quantitatively assess the contribution of multiple variables, including confounding variables and variables of interest, to the results of the study by considering them simultaneously. This approach helped reduce the impact of confounding factors, making the results more reliable and accurate.

#### 2.4.2 Relationship between cheese and fish consumption and depression

In the univariable MR, we estimated the total relationship between cheese and fish consumption and depression as ORs with 95% CIs. To further identify and adjust for possible horizontal pleiotropy biases, we incorporated the MR-PRESSO method into our analysis to detect and correct for outlier SNPs affecting the causal estimates (31). In addition, the impact of individual variants on the associations was evaluated through leave-one-out analyses.

Indian snacks and intake of mango, sushi, and unsalted peanuts were underlying confounding factors for the association between exposures and outcome. Multivariable MR was used to evaluate the relationship between cheese consumption and depression, with adjustment for the aforementioned dietary factors. Because there was no causal relationship between non-oily fish or oily fish consumption and depression, no multivariable MR was conducted.

#### 2.4.3 Mediator screening and two-step MR

**Figure 2:**
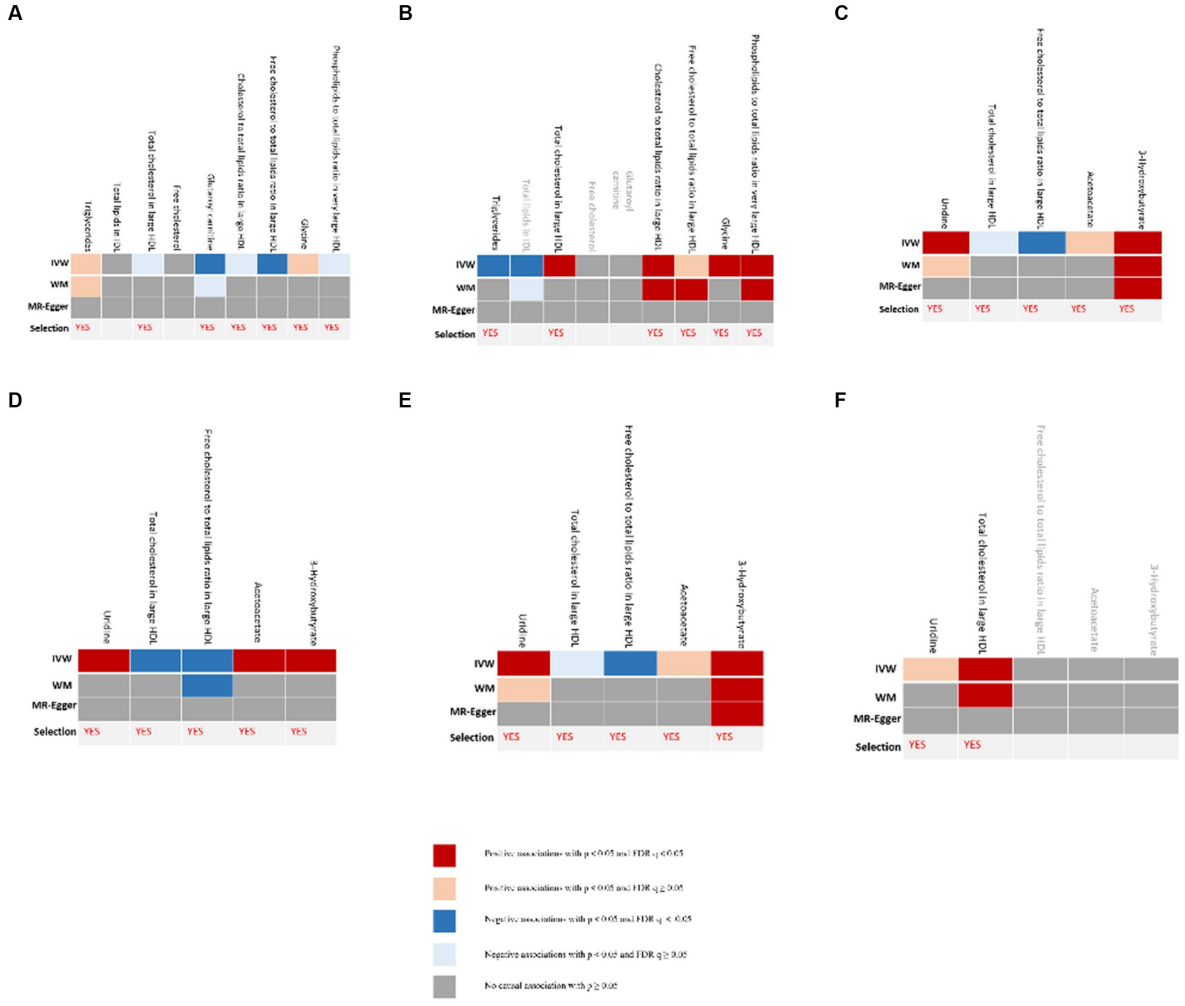
Evidence for selection of mediators that play a role in the association between consumption of cheese and fish and depression. **(A)** Causal associations between candidate mediators and depression in the cheese consumption group; **(B)** Causal associations between cheese consumption and candidate mediators, which were confirmed to influence depression; **(C)** Causal associations between candidate mediators and depression in the non-oily fish consumption group; **(D)** Causal associations between non-oily fish consumption and candidate mediators, which were confirmed to influence depression; **(E)** Causal associations between candidate mediators and depression in the oily fish consumption group; **(F)** Causal associations between oily fish consumption and candidate mediators, which were confirmed to influence depression. “Selection” indicates whether the candidate mediators were selected for subsequent analyses. The IVW method was used for the main analysis. Sensitivity analyses included the MR-Egger and WM shown in the figure as well as the simple mode and weighted mode methods shown in Supplementary Tables S6, S8. FDR, false discovery rate; IVW, inverse-variance weighted.

The process of selecting mediators is shown in Figure 2. We initially utilized univariable MR to investigate the causal link between the 12 possible mediators and depression. We then examined the possible causative association between cheese and fish consumption and possible mediators, which have been confirmed to have a relationship with depression. Following multiple testing, the adjusted *p*-value (*q*-value) was calculated. We categorized IVW outcomes as having substantial evidence if they had *p* < 0.05 and *q* < 0.05, while those with *p* < 0.05 and *q* ≥ 0.05 were considered probable evidence. If there was conclusive proof that cheese and fish consumption affected certain mediators that had an impact on the risk of developing depression, it was these mediators that would be picked.

We conducted two-step MR analyses to evaluate the mediating effects of each selected mediator in the causal pathways linked to cheese and fish consumption and depression. First, we assessed the causal relationship (β1) between cheese and fish consumption on each mediator using univariable MR. Second, we assessed the causal relationship (β2) of every mediator on depression using univariable MR. The mediator relationship between cheese and fish consumption and depression was calculated as follows: β1 × β2 (32).

The MR analyses were performed using R software version 4.2.0 with the packages “TwoSampleMR,” “MRPRESSO,” and “fdrtool.”

### 2.5 Analysis of metabolic pathways

The metabolic pathways were analyzed using Metaboanalyst 5.0 (33).^3^ We identified possible metabolites and pathways that may be related to the biological processes underlying depression by using modules of functional enrichment analyses and pathway analyses.

### 2.6 Molecular docking

We obtained the 3D structure of glycine (PubChem CID: 750) from PubChem.^4^ Using the RCSB Protein Data Bank,^5^ we obtained the structure of receptor proteins. Two receptor proteins were investigated: glutamate receptor 2 (GRIA2, PDB ID: 2WJW) (34) and voltage-dependent L-type calcium channel subunit beta-3 (CACNB3, PDB ID: 7UHG) (35). We used AutoDockTools 1.5.6 to convert positive metabolites (mol2 format) and receptor proteins (pdb format) to pdbqt format (36). Coordinates of active pockets of receptor proteins are listed in Supplementary Table S2. Visualization of docking results was performed using Discovery Studio 2019.

## 3 Results

The United Kingdom Biobank study provided summary statistics on cheese, non-oily fish, and oily fish consumption, with sample sizes of 451,486, 460,880, and 460,443, respectively. Within the Family GWAS Consortium, TG involved 78,700 individuals. Additionally, total cholesterol in large HDL, cholesterol to total lipids ratio in large HDL, free cholesterol to total lipids ratio in large HDL, phospholipids to total lipids ratio in very large HDL, 3-HB, glycine, acetoacetate, and uridine each involved a different number of individuals as follows: 21,558, 115,078, 115,078, 115,053, 113,595, 114,972, 115,075, and 7,800, respectively. In the UK Biobank study, depression was the most tractable phenotype, with SNP associations extracted from a GWAS of 113,769 depression cases and 208,811 controls of European ancestry.

### 3.1 Univariable and multivariable MR analyses assessing the causal association between cheese and fish consumption and depression

**Figure 3:**
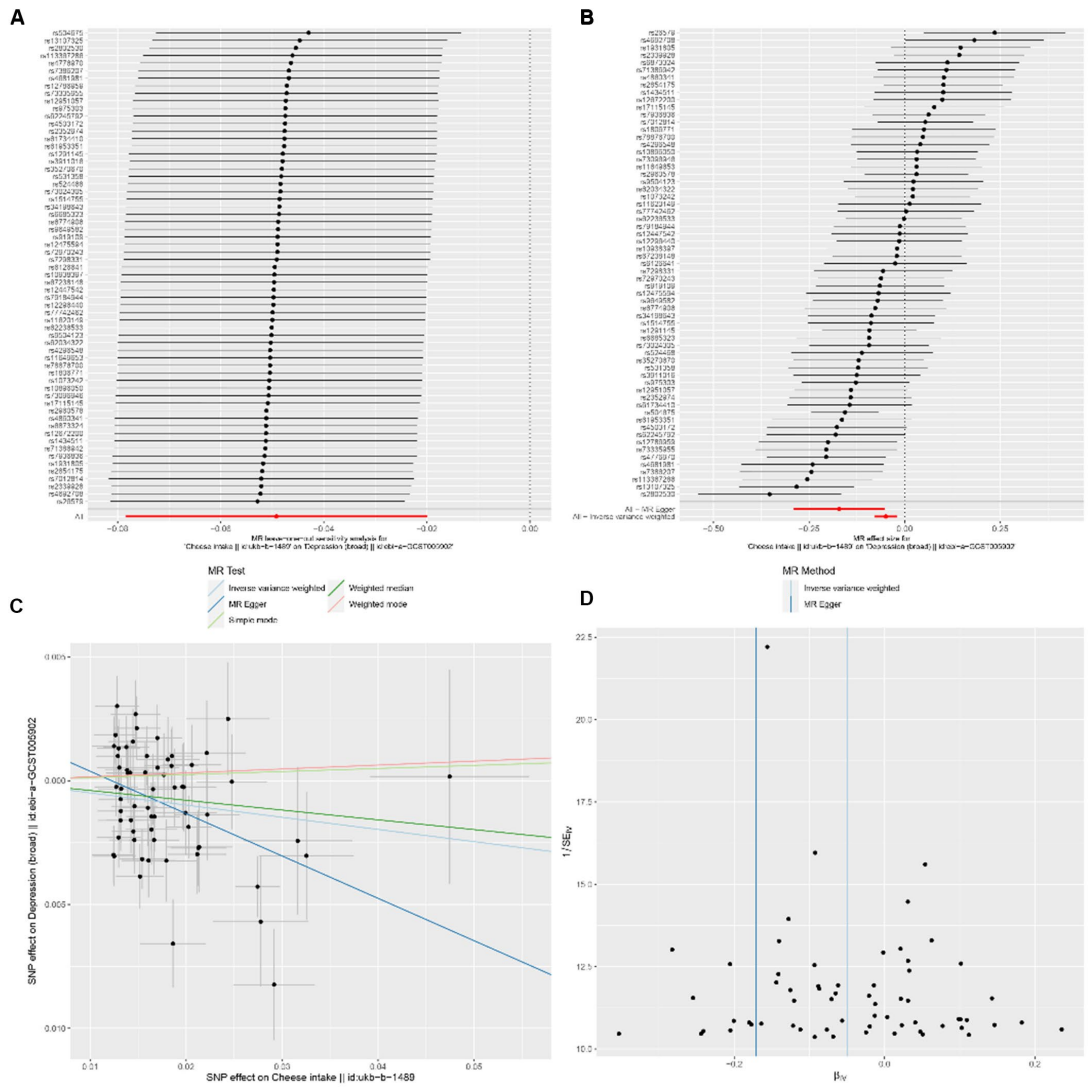
**(A)** Forest plots of leave-one-out analyses for causal SNP relationship between cheese consumption and depression. The error bars indicate the 95% confidence interval (CI). **(C)** Scatter plots for causal SNP relationship between cheese consumption and depression. Each black point represents each SNP on the exposure (horizontal axis) and the outcome (vertical axis) plotted with error bars corresponding to the standard error (SE). The slope of each line corresponds to the combined estimate using each method of the inverse-variance weighted (light blue line), MR-Egger (blue line), simple mode (light green line), weighted median (green line), and weighted mode (pink line). SNP: single nucleotide polymorphism, MR, Mendelian randomization. **(B)** Forest plot to visualize causal effect of each single SNP on depression. **(D)** Funnel plots to visualize overall heterogeneity of MR estimates for the effect of cheese consumption on depression. IVW indicates inverse-variance weighted; and MR, Mendelian randomization.

In the univariable MR analysis, we observed a correlation between the consumption of SD (standard-deviation) cheese and a reduced incidence of depression (IVW-OR: 0.95; 95% CI: 0.92 to 0.98; *p* = 9.86 × 10^−4^). Multiple analyses, employing MR-Egger, weighted median, weighted mode, and simple mode, consistently confirmed the strength and reliability of the IVW results (Supplementary Table S3). For non-oily fish and oily fish consumption, there were no causal relationships with depression risk (*p* = 0.08 and *p* = 0.78, respectively). According to the leave-one-out analysis, no single SNP could be attributed to these findings (Figure 3A). The forest diagram, scatter diagram and funnel diagram were shown in Figures 3B-D, respectively.

The relationship between cheese consumption and depression remained statistically significant in the multivariable MR analyses, even after accounting for consumption of Indian snacks and intake of mango, sushi, and unsalted peanuts, as evidenced by ORs (95% CIs) of 0.95 (0.92 to 0.98), 0.95 (0.93 to 0.98), 0.95 (0.93 to 0.98), and 0.95 (0.92 to 0.98), respectively.

### 3.2 Univariable MR analyses assessing the causal association between possible mediators and depression

Of the 12 possible mediators, it was found that 10 mediators were linked to the risk of depression. There was suggestive evidence for positive causal relationships between TG (OR: 1.01; 95% CI: 1.00 to 1.02; *p* = 0.03), glycine (OR: 1.01; 95% CI: 1.00 to 1.01; *p* = 0.04), uridine (OR: 1.17; 95% CI: 1.02 to 1.34; *p* = 0.02), and depression risk. Total cholesterol in large HDL (OR: 0.99; 95% CI: 0.99 to 1.00; *p* = 0.02), cholesterol to total lipids ratio in large HDL (OR: 0.99; 95% CI: 0.99 to 1.00; *p* = 0.04), and phospholipids to total lipids ratio in very large HDL (OR: 0.99; 95% CI: 0.99 to 1.00; *p* = 0.04) were negatively related to depression.

There was strong evidence for negative causal links between glutaryl carnitine (OR: 0.95; 95% CI: 0.92 to 0.99; *p* = 0.01), free cholesterol to total lipids ratio in large HDL (OR per 1 SD: 0.99; 95% CI: 0.98 to 1.00; *p* = 2.79E-03), and depression, even after adjusting for multiple comparisons using false discovery rate (FDR). Acetoacetate (OR: 1.03; 95% CI: 1.00 to 1.06; *p* = 0.02) and 3-HB (OR: 1.03; 95% CI: 1.01 to 1.05; *p* = 6.22E-04) were positively related to depression risk after adjusting for FDR (Supplementary Table S6). The results did not indicate any evidence of horizontal pleiotropy between possible mediators and depression (intercept ≥ 0.08; Supplementary Table S7).

### 3.3 Univariable MR analyses assessing the causal association between cheese and fish consumption with possible mediators

**Figure 4:**
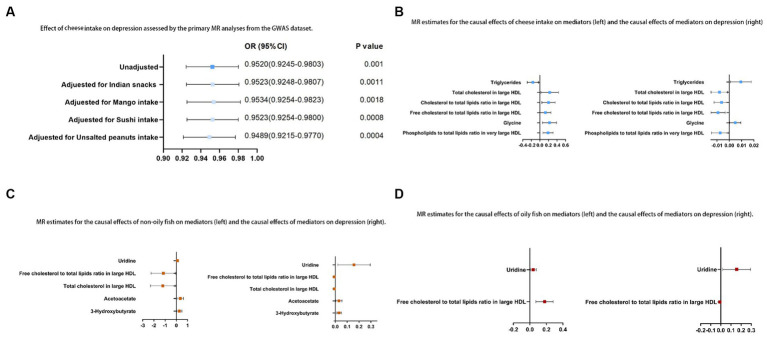
**(A)** Relationship between cheese consumption and depression assessed by the primary MR analyses from the GWAS dataset; **(B)** MR estimates for the causal relationships between cheese consumption and mediators (left) and the causal relationships between mediators and depression (right); **(C)** MR estimates for the causal relationships between non-oily fish consumption and mediators (left) and the causal relationships between mediators and depression (right); **(D)** MR estimates for the causal relationships between oily fish consumption and mediators (left) and the causal relationships between mediators and depression (right).

Of the 10 possible mediators that could impact depression, SD cheese consumption was related to high levels of total cholesterol in large HDL (OR: 1.26; 95% CI: 1.03 to 1.54; *p* = 0.03), cholesterol to total lipids ratio in large HDL (OR: 1.23; 95% CI: 1.05 to 1.43; *p* = 0.01), free cholesterol to total lipids ratio in large HDL (OR: 1.14; 95% CI: 1.00 to 1.29; *p* = 0.04), phospholipids to total lipids ratio in very large HDL (OR: 1.21; 95% CI: 1.08 to 1.37; *p* = 1.28E-03), and glycine (OR: 1.25; 95% CI: 1.06 to 1.47; *p* = 6.71E-03). Cheese consumption was also found to be associated with lower TG (OR: 0.85; 95% CI: 0.75 to 0.97; *p* = 0.01). Each SD increase in non-oily fish consumption was associated with higher levels of uridine (OR: 1.11; 95% CI: 1.01 to 1.22; *p* = 0.04), acetoacetate (OR: 1.42; 95% CI: 1.07 to 1.89; *p* = 0.01), and 3-HB (OR: 1.30; 95% CI: 1.03 to 1.63; *p* = 0.03). Additionally, a decrease in the ratio of free cholesterol to total lipids in large HDL (OR: 0.32; 95% CI: 0.10 to 0.96; *p* = 0.04) and in total cholesterol levels in large HDL (OR: 0.30; 95% CI: 0.1 to 0.9; *p* = 0.03) was observed with non-oily fish consumption. For oily fish consumption, increases in the uridine (OR: 1.04; 95% CI: 1.01 to 1.08; *p* = 0.02) and free cholesterol to total lipids ratio in large HDL (OR: 1.19; 95% CI: 1.08 to 1.33; *p* = 4.0E-03) were observed (Supplementary Table S8). The results described in Sections 3.2 and 3.3 are shown in Figure 4. In the MR analysis, no signs of horizontal pleiotropy were found when examining the relationships between cheese consumption, non-oily fish consumption, oily fish consumption, and potential mediators (Supplementary Table S9).

### 3.4 Two-step MR was employed to estimate the effect of each mediator in moderating the relationship between cheese and fish consumption and depression

**Figure 5:**
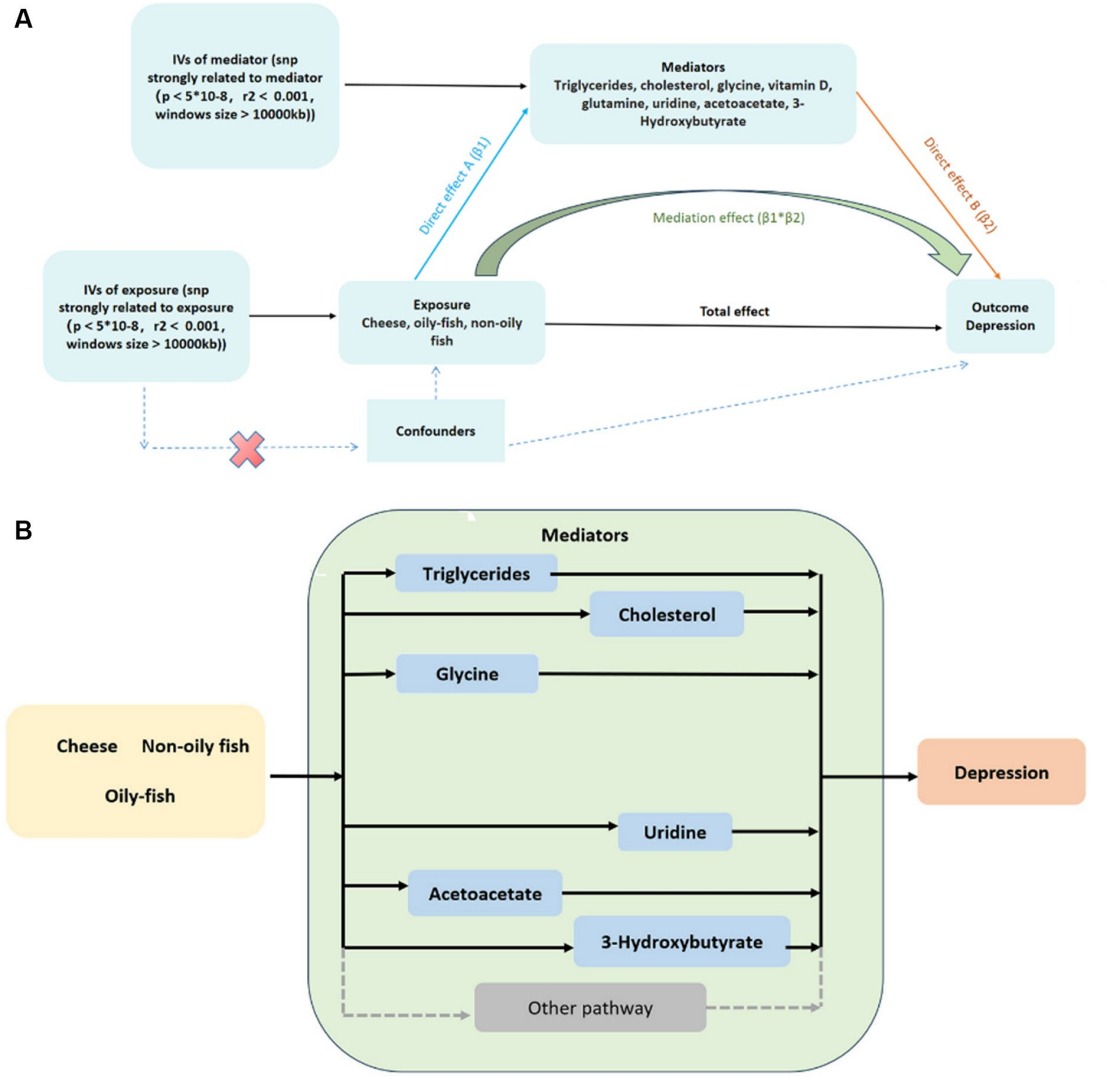
**(A)** Two-step MR analysis framework; **(B)** DAG for the proposed causal interactions for metabolites in the pathway between cheese and fish consumption and depression. The black arrows represent causal pathways from cheese and fish consumption through mediators identified in this MR study to depression. The gray arrows represent other plausible pathways linking cheese and fish consumption to depression that we did not investigate in this study. DAG, directed acyclic diagram; MR, Mendelian randomization.

We employed two-step MR to investigate the possible mediators among nine variables. The mediating effect of TG, total cholesterol in large HDL, cholesterol to total lipids ratio in large HDL, free cholesterol to total lipids ratio in large HDL, glycine, and phospholipids to total lipids ratio in very large HDL of cheese consumption on depression risk were − 0.002 (95% CI: −0.023 - 0.020), −0.002 (95% CI: −0.049 - 0.045), −0.001 (95% CI: −0.033 - 0.031), −0.001 (95% CI: −0.018 - 0.015), 0.001 (95% CI: −0.035 - 0.037), and − 0.001 (95% CI: −0.024 - 0.021), respectively. The mediating effect of uridine, free cholesterol to total lipids ratio in large HDL, total cholesterol in large HDL, acetoacetate, and 3-HB between non-oily fish consumption and depression risk were 0.016 (95% CI: −0.008 - 0.040), 0.011 (95% CI: −1.269 - 1.290), 0.010 (95% CI: −1.316 - 1.335), 0.011 (95% CI: −0.089 - 0.110), and 0.008 (95% CI: −0.051 - 0.068), respectively. Moreover, uridine and free cholesterol to total lipids ratio in large HDL between oily fish consumption and depression risk were 0.006 (95% CI: −0.015- 0.028) and − 0.002 (95% CI: −0.020 - 0.017), respectively (Supplementary Table S10). The interactive causal associations of mediators including lipids, amino acids, uridine, and acetoacetate in the pathway linking cheese and fish consumption and depression are shown in Figure 5.

### 3.5 Metabolic pathway analysis

**Figure 6:**
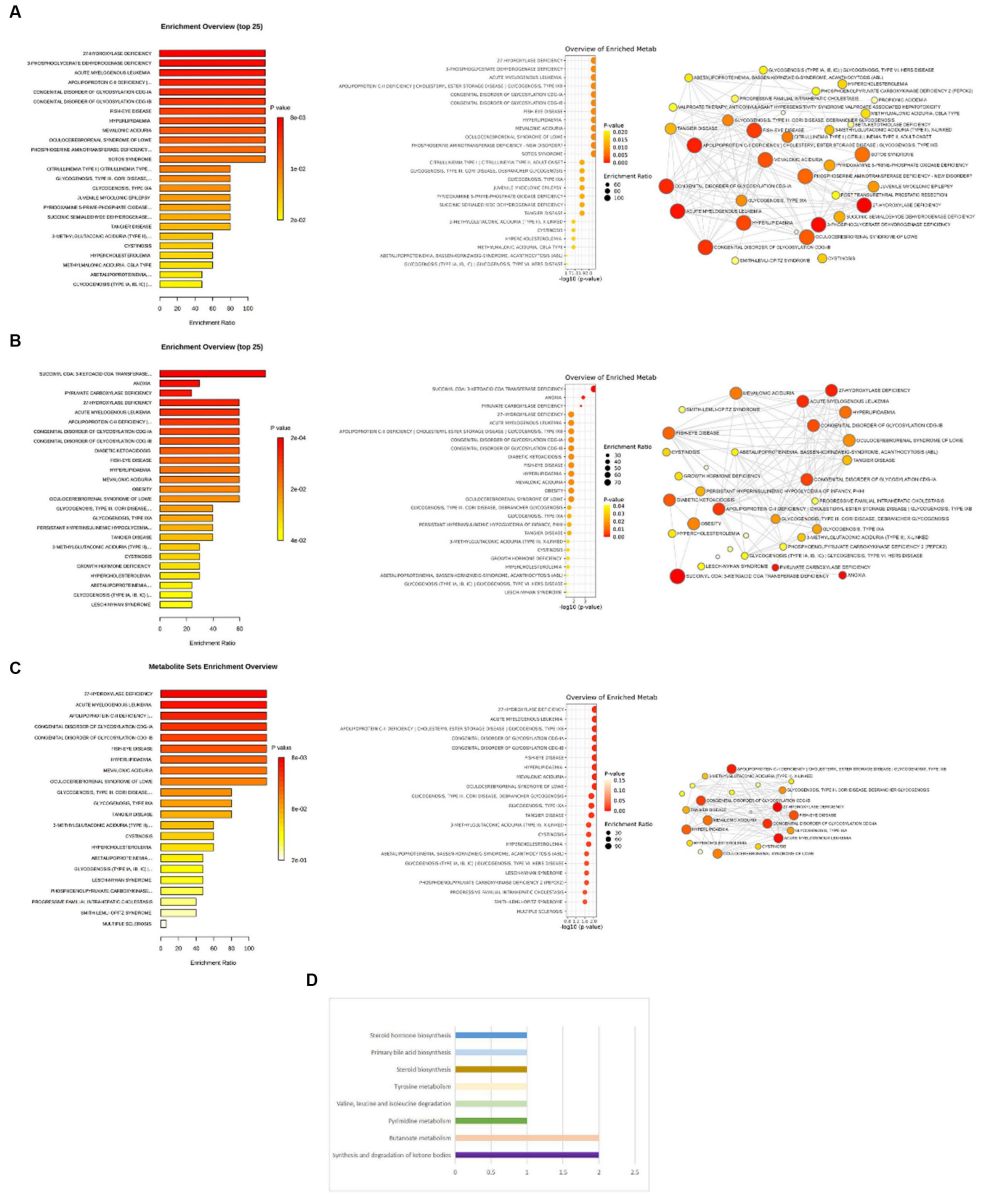
**(A)** Enrichment analysis of pathways related to cheese consumption and the treatment of depression. **(B)** Enrichment analysis of pathways related to non-oily fish consumption and the treatment of depression. **(C)** Enrichment analysis of pathways related to oily fish consumption and the treatment of depression. **(D)** Eight key metabolic pathways related to non-oily fish consumption and the treatment of depression.

The enrichment analysis revealed that 27-hydroxylase deficiency, 3-phosphoglyceratedehydrogenase deficiency, apolipoprotein C-II deficiency cholesteryl ester storage disease glycogenosis, type IXB, acute myelogenous leukemia, congenital disorder of glycosylation CDG-IA, congenital disorder of glycosylation CDG-IB, fish-eye disease, hyperlipidemia, mevalonic aciduria, oculocerebrorenal syndrome of Lowe, phosphoserine aminotransferase deficiency-new disorder, and Sotos syndrome were the enrichment entries most significantly implicated for cheese consumption (Figure 6A). For non-oily fish consumption, succinyl CoA: 3-ketoacid CoA transferase deficiency, anoxia, and pyruvate carboxylase deficiency were the enrichment entries most significantly implicated (Figure 6B). For oily fish consumption, 27-hydroxylase deficiency, acute myelogenous leukemia, apolipoprotein C-II deficiency cholesteryl ester storage disease glycogenosis, type IXB, congenital disorder of glycosylation CDG-IA, congenital disorder of glycosylation CDG-IB, fish eye disease, hyperlipidemia, mevalonic aciduria, and oculocerebrorenal syndrome of Lowe were the enrichment entries most significantly implicated (Figure 6C).

Non-oily fish consumption was found to be correlated with alterations in eight metabolic pathways. Our results show that the “Synthesis and degradation of ketone bodies” (*p* = 4.99E-05), “Butanoate metabolism” (*p* = 5.19E-04), “Pyrimidine metabolism” (*p* = 0.10), “Valine, leucine and isoleucine degradation” (*p* = 0.10), “Tyrosine metabolism” (*p* = 0.10), “Steroid biosynthesis” (*p* = 0.10), “Primary bile acid biosynthesis” (*p* = 0.10), and “Steroid hormone biosynthesis” (*p* = 0.20) have been observed to link the pathogenic process between non-oily fish consumption and depression (Figure 6D).

### 3.6 Molecular docking analysis

**Figure 7:**
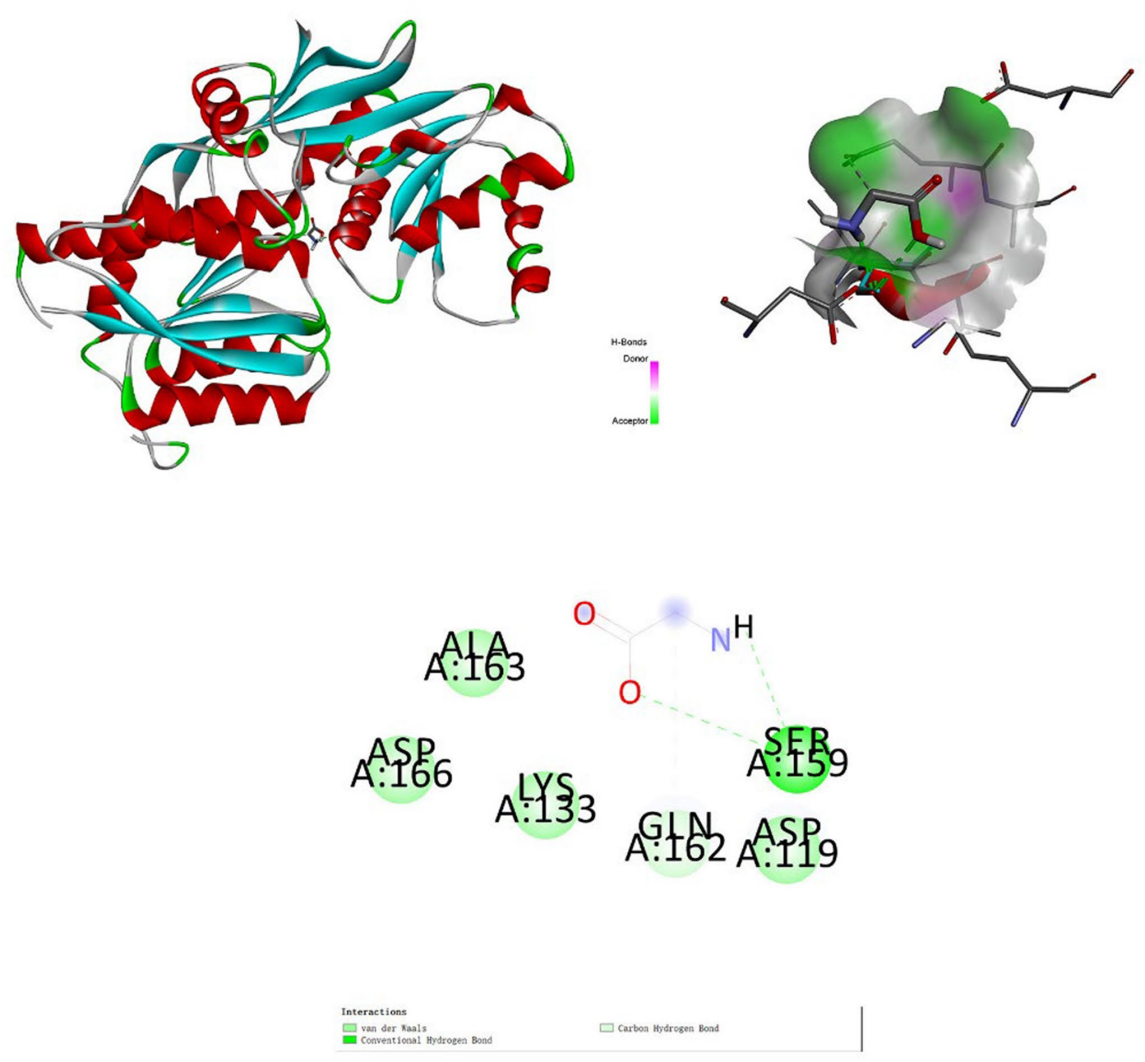
Molecular docking. Binding mode of glycine with glutamate receptor 2.

The association between positive metabolites and receptor proteins was investigated through the application of molecular docking. Using molecular docking, we found the binding energies of glutamate receptor 2 with glycine was −2.9 kcal/mol (RMSD: 1.791) (Figure 7). Glutamate receptor 2 has an important role in depression (37). The docking analysis demonstrated that glycine had a high affinity for the receptor protein for depression.

## 4 Discussion

This study employed an MR analysis to examine the potential causal relationship between cheese and fish consumption and depression. Additionally, the study aimed to identify metabolites that mediated this relationship and examined the pathways connecting cheese and fish consumption with depression. The consumption of cheese showed a negative correlation with depression risk as indicated by genetic instruments. This finding was further substantiated using comprehensive MR analyses. However, there was no direct causal relationship between fish consumption and depression. Of the 12 possible mediators, 9 were found to have a verified role in mediating the causal influence between cheese and fish consumption and depression, involving lipids, amino acids, uridine, and acetoacetate. Our study provided a novel insight into the role of diet in depression pathogenesis and treatment.

The occurrence of depression is shaped by various factors including biology, demographics, genetics, and behavior (38–41). As a potential standalone indicator of disease susceptibility, there has been an increasing emphasis on investigating the influence of habitual dietary intake and specific nutrient consumption (42). Several essential nutrients, including n-3 polyunsaturated fatty acids, B vitamins, magnesium, and zinc, have shown connections to brain function and are currently under investigation as potential distinct indicators of disease risk (40). Nutritional intake may modulate neuronal pathways associated with depression risk, including oxidative stress, neuroplasticity, inflammation, mitochondrial function, and the gut microbiome (40, 43–46). There have been many studies of vitamins, trace elements, or carbohydrates in the previous literature, but no previous study has investigated the causal relationship between cheese and fish consumption and depression and the role of metabolites. In our study, MR approaches were used to minimize possible confounders and subsequently demonstrated a causal relationship between cheese and fish consumption and depression, controlling for other dietary intake. This highlights cheese and fish consumption as important factors for predicting and preventing the risk of depression.

Our study explored the mediators linking cheese and fish consumption and depression. Of the 12 metabolites that encompass lipids, amino acids, nucleotide metabolism, and more, we found TG, cholesterol, 3-HB, glycine, uridine, and acetoacetates may be causal mediators. Elevated triglyceride levels have been correlated with an increase in the production of inflammatory mediators, including tumor necrosis factor-α (TNF-α), interleukin-6 (IL-6), C-reactive protein, and interleukin-1β (IL-1β). These inflammatory mediators are closely associated with the pathogenesis of depression. Furthermore, elevated triglyceride levels have the potential to disrupt the balance of neurotransmitters such as serotonin and dopamine. When these essential brain chemicals deviate from their normal levels, the risk of depression may increase (47). Low serum cholesterol levels may result in a reduced availability of serum-free tryptophan, an amino acid necessary for the synthesis of serotonin in the brain. A reduced supply of tryptophan can lead to reduced serotonin concentrations, a factor associated with depression and suicidal behavior (48). Acetoacetate has been found to protect neurons by promoting the expression of brain-derived neurotrophic factor (BDNF) and inhibiting neuroinflammation in the hippocampus. Miyamoto et al. discovered that acetoacetate acts as a ligand for GPR43 and its coupling inhibits the activation of pERK, as well as its two substrates, IL-6 and TNF-α (49, 50). Massieu et al. conducted both *in vivo* and *in vitro* experiments, demonstrating that acetoacetate protects hippocampal neurons from glutamate-induced neurotoxicity when glycolysis inhibitors are applied (51). In recent studies, it has been suggested that 3-HB may exhibit antidepressant effects by inhibiting the activation of the nucleotide-binding domain, leucine-rich repeat, and pyrin domain-containing 3 (NLRP3) inflammasome (52–54). Yamanishi et al. observed that peripheral administration of 3-HB led to a reduction in inflammatory cytokine levels in the hippocampus, including IL-1β and TNF-α, thereby improving depressive- and anxiety-like behaviors in a rodent model of chronic unpredictable stress (54). Additionally, uridine promotes the synthesis of cytidine 5′-diphosphocholine (CDP-choline), a critical substrate for phospholipid synthesis in the body. Phospholipids are vital constituents of cellular membranes and are responsible for maintaining their integrity and proper functioning. Any disruptions in the synthesis of phospholipids, caused by imbalances in CDP-choline levels influenced by uridine stimulation, may potentially contribute to the manifestation of depressive symptoms. These disturbances can impact the functionality of neurons and neurotransmitter systems that are involved in regulating mood (55). N-methyl-D-aspartate (NMDA) receptors require both glutamate and an auxiliary co-agonist (typically glycine) to be activated. Upon activation, the ion channels of NMDA receptors open, allowing the entry of calcium ions and other ions into the neuron, thereby triggering a series of signal transduction pathways that affect neuronal excitability and neurotransmitter release. By modulating the function of NMDA receptors, glycine can influence neurotransmitter release and neuronal excitability (56, 57).

Over the past few years, the field of metabolomics has become increasingly prominent in diagnosing diseases, studying pathogenesis, identifying drug targets, personalizing drug therapies, and assessing treatment results. It has emerged as a cutting-edge omics platform in medical research (58, 59). Metabolomics serves as an invaluable tool for both physical and mental illness research (60). For instance, comparisons of metabolite concentrations between patients in remission and those currently experiencing depression have revealed similar patterns of metabolite alterations observed in analyses comparing control subjects with individuals currently suffering from depression (58, 61, 62). In our study, nine metabolites and their related pathways were identified to be significantly associated with depression. Molecular docking demonstrated a superior binding affinity between glycine and GRIA2, suggesting that metabolites might play an important role by influencing receptor proteins in the treatment of depression.

One of the benefits of our research was our utilization of MR methods to analyze the relationship and potential mediating factors between cheese and fish consumption and depression. Furthermore, we performed several sensitivity analyses to enhance the precision and reliability of our MR results. Moreover, there were several constraints associated with this research. First, we made the assumption that the relationship between cheese and fish consumption and depression followed a linear pattern in both univariate and multivariate MR analyses. Future studies using data at the individual level should be conducted to explore potential non-linear causal associations. Second, to support clinical practice, we investigated metabolites that were primarily linked to diet or depression as potential mediators. Third, as this study mainly focuses on individuals of European ancestry, future research should focus on other ethnicities.

This study introduced innovative perspectives in the field of nutrition and mental health by examining the relationship between cheese and fish consumption on depression risk. It also shed light on the metabolic regulatory mechanisms influenced by cheese and fish consumption. Additionally, the study proposed personalized prevention and treatment strategies for depression based on individual nutritional characteristics, paving the way for the application of personalized nutritional interventions in the realm of mental health.

## 5 Conclusion

This research showed a clear link between cheese and fish consumption and depression, even after considering factors such as the consumption of Indian snacks, mango, sushi, and unsalted peanuts. It was supported by the findings that dietary choices could influence the occurrence of depression. In addition, lipids, amino acids, uridine, and acetoacetate were identified as causal mediators that play a role in the pathways connecting cheese and fish consumption and depression. Metabolomics research and molecular docking also provided insights into possible metabolic pathways and underlying mechanisms of action. We have provided insight into the pathogenesis of depression as well as possible targets for the prevention, intervention, and prediction of depression.

## Data availability statement

The original contributions presented in the study are included in the article/Supplementary materials, further inquiries can be directed to the corresponding author.

## Author contributions

YC: Formal analysis, Methodology, Software, Visualization, Writing – original draft. JL: Conceptualization, Project administration, Resources, Supervision, Validation, Writing – review & editing. MT: Data curation, Software, Visualization, Writing – review & editing.

## Footnotes

^1^ https://biobank.ctsu.ox.ac.uk/crystal/field.cgi?id=1339

^2^ https://biobank.ctsu.ox.ac.uk/crystal/field.cgi?id=1329

^3^ https://www.metaboanalyst.ca/

^4^ https://www.ncbi.nlm.nih.gov/pccompound/?term

^5^ https://www.rcsb.org/
