# Supplementary material for: Association between cheese and fish consumption and the occurrence of depression based on European population: mediating role of metabolites
Source: Front Nutr. 2024 Apr 17;11:1322254. doi: 10.3389/fnut.2024.1322254 (PMC11061354; doi:10.3389/fnut.2024.1322254)
Supplement: Supplementary file 1 [file Table_1.DOCX]

Supplementary Material

Table 1 GWAS data sources of the MR study

| **Phenotype** | **PMID or GWAS ID** | **Sample size or number of SNPs** | **Ancestry** |
| --- | --- | --- | --- |
| Exposure  Cheese intake | ukb-b-1489 | 451,486 | European |
| Mediator  Triglycerides  Total cholesterol in large HDL  Cholesterol to total lipids ratio in large HDL  Free cholesterol to total lipids ratio in large HDL  Glycine  Phospholipids to total lipids ratio in very large HDL | ieu-b-4850  27005778  met-d-L_HDL_C_pct  met-d-L_HDL_FC_pct  met-d-Gly  met-d XL_HDL_PL_pct | 78,700  21,558  115,078  115,078  114,972  115,053 | European  European  European  European  European  European |
| Exposure  Non-oily fish intake | ukb-b-17627 | 460,880 | European |
| Mediator  Uridine  Free cholesterol to total lipids ratio in large HDL  Total cholesterol in large HDL  Acetoacetate  3-Hydroxybutyrate | 24816252  met-d-L_HDL_FC_pct  27005778  met-d-Acetoacetate  met-d-bOHbutyrate | 7,800  115,078  21,558  115,075  113,595 | European  European  European  European  European |
| Exposure  Oily fish intake | ukb-b-2209 | 460,443 | European |
| Mediator  Uridine  Free cholesterol to total lipids ratio in large HDL | 24816252  met-d-L_HDL_FC_pct | 7,800  115,078 | European  European |
| Outcome  Depression | 29662059 | 322,580 | European |

**Table S2. Coordinates of active pockets of receptor proteins.**

| Glutamate Receptor 2 | | |
| --- | --- | --- |
| --center_x -41.9 | --center_y -28.8 | --center_z -14.1 |
| --size_x 19.4 | --size_y 30.2 | --size_z 36.2 |
| Voltage-dependent L-type calcium channel subunit beta-3 | | |
| --center_x 164.3 | --center_y 169.3 | --center_z 156.2 |
| --size_x 71.7 | --size_y 39.0 | --size_z 46.7 |

**Table S3. MR results for the causal effect of cheese intake on depression**

| **Depression data source** | **Method** | **No. of SNPs** | **OR (95% CI)**^†^ | **P value** |
| --- | --- | --- | --- | --- |
| Depression | IVW | 63 | 0.95 (0.92-0.98) | 9.86E-04 |
|  | MR Egger |  | 0.84 (0.75-0.95) | 6.4E-03 |
|  | Simple mode |  | 1.01 (0.92-1.11) | 0.80 |
|  | Weighted median |  | 0.96 (0.93-1.00) | 0.03 |
|  | Weighted mode |  | 1.02 (0.91-1.14) | 0.78 |

OR (95% CI) represents risks for depression associated with each SD added with diets.

Abbreviations: CI=confidence interval; IVW=inverse variance weighted; MR=Mendelian randomization; No=number; OR=odds ratio; SNP=single nucleotide polymorphism.

**Table S4. Univariable MR pleiotropy and heterogeneity test for the association between cheese intake and depression**

| **Horizontal pleiotropy** | | | | | |
| --- | --- | --- | --- | --- | --- |
| **Exposure** | **Outcome** | **Method** | **Egger_intercept** | **Intercept_se** | **P_intercept_** |
| Cheese intake | Depression | MR Egger | 2.09E-03 | 1.01E-03 | 0.04 |
| **Heterogeneity test** | | | | | |
| **Exposure** | **Outcome** | **Method** | **Q statistic** | **Q_df** | **P_heterogeneity_** |
| Cheese intake | Depression | IVW | 122.86 | 62 | 6.74E-06 |
|  |  | MR Egger | 114.78 | 61 | 3.78E-05 |

Abbreviations: IVW=inverse variance weighted; MR=Mendelian randomization.

**Table S5. Multivariable MR estimates for the independent effect of cheese intake on depression with adjustment for other dietary intake.**

| **Variable** | **Method** | **OR (95% CI)†** | **P value** |
| --- | --- | --- | --- |
| ***Depression*** | | | |
| Cheese intake | MV-IVW | 0.95 (0.92-0.98) | 1.09E-03 |
| Indian snacks |  | 0.98 (0.82-1.18) | 0.83 |
| Cheese intake | MV-IVW | 0.95 (0.93-0.98) | 1.76E-03 |
| Mango intake |  | 0.94 (0.77-1.14) | 0.52 |
| Cheese intake | MV-IVW | 0.95 (0.93-0.98) | 8.32E-04 |
| Sushi intake |  | 1.22 (0.92-1.63) | 0.17 |
| Cheese intake | MV-IVW | 0.95 (0.92-0.98) | 4.29E-04 |
| Unsalted peanuts intke |  | 1.16 (0.88-1.54) | 0.28 |

**Table S6. Univariable MR estimates for the causal effects of potential mediators on depression**

| **Exposure** | **Method** | **No. of SNP** | **OR (95% CI)**^†^ | **P value** | **FDR q-value** |
| --- | --- | --- | --- | --- | --- |
| Triglycerides | IVW | 37 | 1.01 (1.00-1.02) | 0.03 | 0.06 |
|  | MR Egger |  | 1.01 (1.00-1.03) | 0.09 | NA |
|  | Simple mode |  | 1.01 (0.99-1.03) | 0.36 | NA |
|  | Weighted median |  | 1.01 (1.00-1.03) | 0.02 | NA |
|  | Weighted mode |  | 1.01 (1.00-1.03) | 0.07 | NA |
| Total lipids in IDL | IVW | 20 | 1.00 (0.99-1.00) | 0.14 | 0.14 |
|  | MR Egger |  | 0.99 (0.98-1.00) | 0.10 | NA |
|  | Simple mode |  | 0.99 (0.98-1.01) | 0.35 | NA |
|  | Weighted median |  | 1.00 (0.99-1.00) | 0.19 | NA |
|  | Weighted mode |  | 0.99 (0.99-1.00) | 0.17 | NA |
| Total cholesterol in large HDL | IVW | 15 | 0.99 (0.99-1.00) | 0.02 | 0.06 |
|  | MR Egger |  | 1.00 (0.98-1.01) | 0.83 | NA |
|  | Simple mode |  | 1.00 (0.98-1.01) | 0.61 | NA |
|  | Weighted median |  | 1.00 (0.99-1.00) | 0.32 | NA |
|  | Weighted mode |  | 1.00 (0.99-1.01) | 0.60 | NA |
| Free cholesterol | IVW | 11 | 0.99 (0.99-1.00) | 0.09 | 0.11 |
|  | MR Egger |  | 0.99 (0.98-1.00) | 0.13 | NA |
|  | Simple mode |  | 1.00 (0.98-1.01) | 0.51 | NA |
|  | Weighted median |  | 0.99 (0.99-1.00) | 0.15 | NA |
|  | Weighted mode |  | 0.99 (0.99-1.00) | 0.28 | NA |
| Glutaroyl carnitine | IVW | 8 | 0.95 (0.92-0.99) | 0.01 | 0.03 |
|  | MR Egger |  | 0.95 (0.81-1.12) | 0.57 | NA |
|  | Simple mode |  | 0.93 (0.87-1.00) | 0.10 | NA |
|  | Weighted median |  | 0.94 (0.90-0.98) | 0.01 | NA |
|  | Weighted mode |  | 0.93 (0.88-0.99) | 0.04 | NA |
| Cholesterol to total lipids ratio in large HDL | IVW | 75 | 0.99 (0.99-1.00) | 0.04 | 0.06 |
|  | MR Egger |  | 0.99 (0.98-1.00) | 0.10 | NA |
|  | Simple mode |  | 0.99 (0.97-1.01) | 0.30 | NA |
|  | Weighted median |  | 0.99 (0.98-1.00) | 0.16 | NA |
|  | Weighted mode |  | 0.99 (0.99-1.00) | 0.16 | NA |
| Free cholesterol to total lipids ratio in large HDL | IVW | 66 | 0.99 (0.98-1.00) | 2.79E-03 | 0.03 |
|  | MR Egger |  | 0.99 (0.98-1.00) | 0.05 | NA |
|  | Simple mode |  | 0.99 (0.97-1.00) | 0.09 | NA |
|  | Weighted median |  | 0.99 (0.98-1.00) | 0.14 | NA |
|  | Weighted mode |  | 0.99 (0.98-1.00) | 0.07 | NA |
| Phospholipids to total lipids ratio in very large HDL | IVW | 63 | 0.99 (0.99-1.00) | 0.04 | 0.06 |
|  | MR Egger |  | 0.99 (0.98-1.00) | 0.19 | NA |
|  | Simple mode |  | 1.00 (0.98-1.02) | 0.83 | NA |
|  | Weighted median |  | 0.99 (0.98-1.00) | 0.15 | NA |
|  | Weighted mode |  | 0.99 (0.99-1.00) | 0.19 | NA |
| Glycine | IVW | 41 | 1.01 (1.00-1.01) | 0.04 | 0.06 |
|  | MR Egger |  | 1.01 (1.00-1.01) | 0.40 | NA |
|  | Simple mode |  | 1.00 (0.98-1.02) | 0.99 | NA |
|  | Weighted median |  | 1.00 (0.99-1.01) | 0.08 | NA |
|  | Weighted mode |  | 1.00 (0.99-1.01) | 0.13 | NA |
| Uridine | IVW | 3 | 1.17 (1.02-1.34) | 0.02 | 0.09 |
|  | MR Egger |  | 1.32 (0.53-3.28) | 0.66 | NA |
|  | Simple mode |  | 1.23 (1.00-1.52) | 0.19 | NA |
|  | Weighted median |  | 1.18 (1.01-1.39) | 0.04 | NA |
|  | Weighted mode |  | 1.21 (1.00-1.47) | 0.19 | NA |
| Acetoacetate | IVW | 7 | 1.03 (1.00-1.06) | 0.02 | 0.02 |
|  | MR Egger |  | 1.04 (0.97-1.12) | 0.32 | NA |
|  | Simple mode |  | 1.04 (0.99-1.09) | 0.15 | NA |
|  | Weighted median |  | 1.03 (1.00-1.06) | 0.09 | NA |
|  | Weighted mode |  | 1.03 (0.98-1.07) | 0.32 | NA |
| 3-Hydroxybutyrate | IVW | 16 | 1.03 (1.01-1.05) | 6.22E-04 | 2.1E-03 |
|  | MR Egger |  | 1.07 (1.02-1.12) | 0.01 | NA |
|  | Simple mode |  | 1.04 (1.00-1.08) | 0.07 | NA |
|  | Weighted median |  | 1.04 (1.01-1.07) | 1.92E-03 | NA |
|  | Weighted mode |  | 1.04 (1.00-1.08) | 0.03 | NA |

**Table S7. Univariable MR pleiotropy and heterogeneity test for the associations between potential mediators and depression**

| **Horizontal pleiotropy test** | | | | |
| --- | --- | --- | --- | --- |
| **Exposure** | **Method** | **Egger_intercept** | **Intercept_se** | **P_intercept_** |
| Triglycerides | MR Egger | -3.78E-04 | 4.98E-04 | 0.45 |
| Total lipids in IDL | MR Egger | 5.78E-04 | 5.87E-04 | 0.34 |
| Total cholesterol in large HDL | MR Egger | -9.31E-04 | 1.13E-03 | 0.42 |
| Free cholesterol | MR Egger | 1.01E-03 | 1.02E-03 | 0.35 |
| Glutaroyl carnitine | MR Egger | -1.07E-07 | 2.18E-03 | 1.00 |
| Cholesterol to total lipids ratio in large HDL | MR Egger | 1.45E-04 | 2.78E-04 | 0.60 |
| Free cholesterol to total lipids ratio in large HDL | MR Egger | 2.78E-05 | 2.88E-04 | 0.92 |
| Phospholipids to total lipids ratio in very large HDL | MR Egger | 1.64E-05 | 3.14E-04 | 0.96 |
| Glycine | MR Egger | 5.03E-04 | 2.78E-04 | 0.08 |
| Uridine | MR Egger | -1.33E-03 | 5.14E-03 | 0.84 |
| Acetoacetate | MR Egger | -5.41E-04 | 1.76E-03 | 0.77 |
| 3-Hydroxybutyrate | MR Egger | -1.71E-03 | 1.05E-03 | 0.13 |
| **Heterogeneity test** | | | | |
| **Exposure** | **Method** | **Q statistic** | **Q_df** | **P_heterogeneity_** |
| Triglycerides | IVW | 41.88 | 36 | 0.23 |
|  | MR Egger | 41.20 | 35 | 0.22 |
| Total lipids in IDL | IVW | 14.62 | 19 | 0.75 |
|  | MR Egger | 13.65 | 18 | 0.75 |
| Total cholesterol in large HDL | IVW | 18.03 | 14 | 0.21 |
|  | MR Egger | 17.13 | 13 | 0.19 |
| Free cholesterol | IVW | 4.49 | 10 | 0.92 |
|  | MR Egger | 3.50 | 9 | 0.94 |
| Glutaroyl carnitine | IVW | 7.27 | 7 | 0.40 |
|  | MR Egger | 7.27 | 6 | 0.30 |
| Cholesterol to total lipids ratio in large HDL | IVW | 77.69 | 74 | 0.36 |
|  | MR Egger | 77.40 | 73 | 0.34 |
| Free cholesterol to total lipids ratio in large HDL | IVW | 67.96 | 65 | 0.38 |
|  | MR Egger | 67.95 | 64 | 0.34 |
| Phospholipids to total lipids ratio in very large HDL | IVW | 75.61 | 62 | 0.11 |
|  | MR Egger | 75.60 | 61 | 0.10 |
| Glycine | IVW | 48.22 | 40 | 0.17 |
|  | MR Egger | 44.50 | 39 | 0.25 |
| Uridine | IVW | 2.18 | 2 | 0.34 |
|  | MR Egger | 2.04 | 1 | 0.15 |
| Acetoacetate | IVW | 2.64 | 6 | 0.85 |
|  | MR Egger | 2.54 | 5 | 0.77 |
| 3-Hydroxybutyrate | IVW | 17.23 | 15 | 0.31 |
|  | MR Egger | 14.48 | 14 | 0.41 |

IVW=inverse variance weighted; MR=Mendelian randomization.

**Table S8. Univariable MR estimates for the causal effect of cheese intake and fish intake on potential mediators**

| **Univariable MR estimates for the causal effect of cheese intake on potential mediators** | | | | | |
| --- | --- | --- | --- | --- | --- |
| **Exposure** | **Method** | **No. of SNP** | **OR (95% CI)**^†^ | **P value** | **FDR q-value** |
| Triglycerides | IVW | 51 | 0.85 (0.75-0.97) | 0.01 | 0.03 |
|  | MR Egger |  | 0.90 (0.53-1.54) | 0.70 | NA |
|  | Simple mode |  | 0.93 (0.62-1.39) | 0.74 | NA |
|  | Weighted median |  | 0.92 (0.79-1.07) | 0.27 | NA |
|  | Weighted mode |  | 1.03 (0.73-1.47) | 0.85 | NA |
| Total lipids in IDL | IVW | 64 | 0.78 (0.63- 0.96) | 0.02 | 0.04 |
|  | MR Egger |  | 1.16 (0.46-2.92) | 0.75 | NA |
|  | Simple mode |  | 0.76 (0.40-1.47) | 0.43 | NA |
|  | Weighted median |  | 0.74 (0.57-0.97) | 0.03 | NA |
|  | Weighted mode |  | 0.70 (0.35-1.40) | 0.32 | NA |
| Total cholesterol in large HDL | IVW | 64 | 1.26 (1.03-1.54) | 0.03 | 0.04 |
|  | MR Egger |  | 1.23 (0.50-3.02) | 0.66 | NA |
|  | Simple mode |  | 1.02 (0.56-1.86) | 0.95 | NA |
|  | Weighted median |  | 1.07 (0.82-1.41) | 0.61 | NA |
|  | Weighted mode |  | 1.05 (0.63-1.76) | 0.85 | NA |
| Free cholesterol | IVW | 64 | 0.79 (0.60-1.06) | 0.11 | 0.11 |
|  | MR Egger |  | 0.95 (0.28-3.25) | 0.93 | NA |
|  | Simple mode |  | 0.94 (0.39-2.24) | 0.89 | NA |
|  | Weighted median |  | 0.82 (0.58-1.15) | 0.25 | NA |
|  | Weighted mode |  | 0.81 (0.39-1.65) | 0.56 | NA |
| Glutaroyl carnitine | IVW | 44 | 1.03 (0.98-1.09) | 0.24 | 0.23 |
|  | MR Egger |  | 1.05 (0.80-1.37) | 0.73 | NA |
|  | Simple mode |  | 1.01 (0.84-1.22) | 0.93 | NA |
|  | Weighted median |  | 1.02 (0.94-1.11) | 0.59 | NA |
|  | Weighted mode |  | 1.03 (0.85-1.23) | 0.79 | NA |
| Cholesterol to total lipids ratio in large HDL | IVW | 64 | 1.23 (1.05-1.43) | 0.01 | 0.03 |
|  | MR Egger |  | 1.15 (0.60-2.24) | 0.67 | NA |
|  | Simple mode |  | 1.18 (0.85-1.63) | 0.33 | NA |
|  | Weighted median |  | 1.21 (1.07-1.37) | 2.4E-03 | NA |
|  | Weighted mode |  | 1.10 (0.82-1.48) | 0.53 | NA |
| Free cholesterol to total lipids ratio in large HDL | IVW | 64 | 1.14 (1.00-1.29) | 0.04 | 0.05 |
|  | MR Egger |  | 1.09 (0.64-1.87) | 0.75 | NA |
|  | Simple mode |  | 1.24 (0.94-1.64) | 0.13 | NA |
|  | Weighted median |  | 1.14 (1.02-1.27) | 0.02 | NA |
|  | Weighted mode |  | 1.03 (0.82-1.29) | 0.79 | NA |
| Phospholipids to total lipids ratio in very large HDL | IVW | 64 | 1.21 (1.08-1.37) | 1.28E-03 | 0.01 |
|  | MR Egger |  | 1.17 (0.71-1.93) | 0.54 | NA |
|  | Simple mode |  | 1.13 (0.86-1.47) | 0.38 | NA |
|  | Weighted median |  | 1.18 (1.06-1.32) | 3.06E-03 | NA |
|  | Weighted mode |  | 1.10 (0.88-1.39) | 0.40 | NA |
| Glycine | IVW | 64 | 1.25 (1.06-1.47) | 6.71E-03 | 0.03 |
|  | MR Egger |  | 1.51 (0.76-3.01) | 0.24 | NA |
|  | Simple mode |  | 1.07 (0.85-1.35) | 0.58 | NA |
|  | Weighted median |  | 1.09 (0.97-1.22) | 0.14 | NA |
|  | Weighted mode |  | 1.06 (0.88-1.28) | 0.56 | NA |
| **Univariable MR estimates for the causal effect of non-oily fish intake on potential mediators** | | | | | |
| **Exposure** | **Method** | **No. of SNP** | **OR (95% CI)**^†^ | **P value** | **FDR q-value** |
| Uridine | IVW | 9 | 1.11 (1.01-1.22) | 0.04 | 0.04 |
|  | MR Egger |  | 1.35 (0.81-2.25) | 0.29 | NA |
|  | Simple mode |  | 1.09 (0.93-1.29) | 0.32 | NA |
|  | Weighted median |  | 1.09 (0.97-1.23) | 0.14 | NA |
|  | Weighted mode |  | 1.09 (0.92-1.29) | 0.33 | NA |
| Free cholesterol to total lipids ratio in large HDL | IVW | 11 | 0.32 (0.10- 0.96) | 0.04 | 0.04 |
|  | MR Egger |  | 2.51 (0.01-532.40) | 0.74 | NA |
|  | Simple mode |  | 0.75 (0.50-1.13) | 0.20 | NA |
|  | Weighted median |  | 0.59 (0.42-0.83) | 2.2E-03 | NA |
|  | Weighted mode |  | 0.63 (0.43-0.93) | 0.04 | NA |
| Total cholesterol in large HDL | IVW | 10 | 0.30 (0.10-0.9) | 0.03 | 0.04 |
|  | MR Egger |  | 2.09 (9.28E-03-468.93) | 0.80 | NA |
|  | Simple mode |  | 0.53 (0.15-1.92) | 0.36 | NA |
|  | Weighted median |  | 0.49 (0.20-1.17) | 0.11 | NA |
|  | Weighted mode |  | 0.65 (0.21-1.99) | 0.47 | NA |
| Acetoacetate | IVW | 11 | 1.42 (1.07-1.89) | 0.01 | 0.04 |
|  | MR Egger |  | 1.05 (0.26-4.26) | 0.94 | NA |
|  | Simple mode |  | 1.13 (0.72-1.77) | 0.61 | NA |
|  | Weighted median |  | 1.25 (0.92-1.70) | 0.15 | NA |
|  | Weighted mode |  | 1.22 (0.82-1.81) | 0.35 | NA |
| 3-Hydroxybutyrate | IVW | 11 | 1.30 (1.03-1.63) | 0.03 | 0.04 |
|  | MR Egger |  | 1.81 (0.59-5.55) | 0.32 | NA |
|  | Simple mode |  | 1.09 (0.65-1.83) | 0.74 | NA |
|  | Weighted median |  | 1.15 (0.84-1.56) | 0.38 | NA |
|  | Weighted mode |  | 1.11 (0.71-1.74) | 0.66 | NA |
| **Univariable MR estimates for the causal effect of oily fish intake on potential mediators** | | | | | |
| **Exposure** | **Method** | **No. of SNP** | **OR (95% CI)**^†^ | **P value** | **FDR q-value** |
| Uridine | IVW | 48 | 1.04 (1.01-1.08) | 0.02 | 0.05 |
|  | MR Egger |  | 1.08 (0.93-1.26) | 0.31 | NA |
|  | Simple mode |  | 1.02 (0.91-1.14) | 0.70 | NA |
|  | Weighted median |  | 1.03 (0.98-1.08) | 0.26 | NA |
|  | Weighted mode |  | 1.04 (0.95-1.13) | 0.40 | NA |
| Free cholesterol to total lipids ratio in large HDL | IVW | 62 | 1.19 (1.08-1.33) | 8.06E-04 | 4.0E-03 |
|  | MR Egger |  | 0.76 (0.51-1.15) | 0.20 | NA |
|  | Simple mode |  | 1.31 (0.98-1.76) | 0.07 | NA |
|  | Weighted median |  | 1.24 (1.10-1.40) | 5.94E-04 | NA |
|  | Weighted mode |  | 1.36 (1.02-1.81) | 0.04 | NA |
| Total cholesterol in large HDL | IVW | 59 | 0.90 (0.70- 1.16) | 0.41 | 0.41 |
|  | MR Egger |  | 0.46 (0.16- 1.35) | 0.17 | NA |
|  | Simple mode |  | 0.77 (0.39- 1.55) | 0.47 | NA |
|  | Weighted median |  | 0.84 (0.61- 1.14) | 0.26 | NA |
|  | Weighted mode |  | 0.73 (0.41- 1.30) | 0.29 | NA |
| Acetoacetate | IVW | 62 | 1.09 (0.99- 1.21) | 0.07 | 0.10 |
|  | MR Egger |  | 1.38 (0.93- 2.05) | 0.11 | NA |
|  | Simple mode |  | 1.02 (0.75- 1.40) | 0.88 | NA |
|  | Weighted median |  | 1.04 (0.92- 1.18) | 0.51 | NA |
|  | Weighted mode |  | 1.03 (0.78- 1.36) | 0.83 | NA |
| 3-Hydroxybutyrate | IVW | 61 | 1.08 (0.99- 1.19) | 0.08 | 0.10 |
|  | MR Egger |  | 1.31 (0.90- 1.90) | 0.16 | NA |
|  | Simple mode |  | 1.12 (0.80- 1.55) | 0.51 | NA |
|  | Weighted median |  | 1.08 (0.96 - 1.22) | 0.19 | NA |
|  | Weighted mode |  | 1.09 (0.81- 1.46) | 0.56 | NA |

OR (95% CI) ^†^ represents the associations of each SD diet addad with each potential mediator.

Abbreviations: CI=confidence interval; FDR=false discovery rate; IVW=inverse variance weighted; MR=Mendelian randomization; NA=not applicable; No=number; SNP=single nucleotide polymorphism.

**Table S9. Univariable MR pleiotropy and heterogeneity test for the associations between chesse intake and potential mediators**

| **Horizontal pleiotropy test** | | | | |
| --- | --- | --- | --- | --- |
| **Exposure** | **Method** | **Egger_intercept** | **Intercept_se** | **P_intercept_** |
| Triglycerides | MR Egger | -1.00E-03 | 4.55E-03 | 0.83 |
| Total lipids in IDL | MR Egger | -6.85E-03 | 7.78E-03 | 0.38 |
| Total cholesterol in large HDL | MR Egger | 4.4E-04 | 7.59E-03 | 0.95 |
| Free cholesterol | MR Egger | -3.03E-03 | 0.01 | 0.77 |
| Glutaroyl carnitine | MR Egger | -3E-04 | 2.21E-03 | 0.89 |
| Cholesterol to total lipids ratio in large HDL | MR Egger | 1.05E-03 | 5.62E-04 | 0.85 |
| Free cholesterol to total lipids ratio in large HDL | MR Egger | 6.83E-04 | 4.57E-03 | 0.88 |
| Phospholipids to total lipids ratio in very large HDL | MR Egger | 6.2E-04 | 4.24E-03 | 0.88 |
| Glycine | MR Egger | -3.27E-03 | 5.83E-03 | 0.58 |
| **Heterogeneity test** | | | | |
| **Exposure** | **Method** | **Q statistic** | **Q_df** | **P_heterogeneity_** |
| Triglycerides | IVW | 90.21 | 50 | 4.27E-04 |
|  | MR Egger | 90.12 | 49 | 3.13E-04 |
| Total lipids in IDL | IVW | 86.19 | 63 | 0.03 |
|  | MR Egger | 85.13 | 62 | 0.03 |
| Total cholesterol in large HDL | IVW | 84.02 | 63 | 0.04 |
|  | MR Egger | 84.01 | 62 | 0.03 |
| Free cholesterol | IVW | 107.12 | 63 | 4.43E-04 |
|  | MR Egger | 106.97 | 62 | 3.41E-04 |
| Glutaroyl carnitine | IVW | 39.54 | 43 | 0.62 |
|  | MR Egger | 39.52 | 42 | 0.58 |
| Cholesterol to total lipids ratio in large HDL | IVW | 329.51 | 63 | 9.61E-38 |
|  | MR Egger | 329.32 | 62 | 4.46E-38 |
| Free cholesterol to total lipids ratio in large HDL | IVW | 223.87 | 63 | 7.07E-20 |
|  | MR Egger | 223.79 | 62 | 3.79E-20 |
| Phospholipids to total lipids ratio in very large HDL | IVW | 197.51 | 63 | 8.61E-16 |
|  | MR Egger | 197.44 | 62 | 4.89E-16 |
| Glycine | IVW | 354.61 | 63 | 3.15E-42 |
|  | MR Egger | 352.81 | 62 | 2.75E-42 |

**Univariable MR pleiotropy and heterogeneity test for the associations between non-oily fish intake and potential mediators**

| **Horizontal pleiotropy test** | | | | |
| --- | --- | --- | --- | --- |
| **Exposure** | **Method** | **Egger_intercept** | **Intercept_se** | **P_intercept_** |
| Uridine | MR Egger | -2.43E-03 | 3.17E-03 | 0.47 |
| Free cholesterol to total lipids ratio in large HDL | MR Egger | -0.03 | 0.03 | 0.46 |
| Total cholesterol in large HDL | MR Egger | -0.02 | 0.03 | 0.49 |
| Acetoacetate | MR Egger | 3.74E-03 | 8.72E-03 | 0.68 |
| 3-Hydroxybutyrate | MR Egger | -6.33E-04 | 2.76E-03 | 0.82 |
| **Heterogeneity test** | | | | |
| **Exposure** | **Method** | **Q statistic** | **Q_df** | **P_heterogeneity_** |
| Uridine | IVW | 3.27 | 8 | 0.92 |
|  | MR Egger | 2.68 | 7 | 0.91 |
| Free cholesterol to total lipids ratio in large HDL | IVW | 282.64 | 10 | 7.22E-55 |
|  | MR Egger | 264.89 | 9 | 7.13E-52 |
| Total cholesterol in large HDL | IVW | 32.09 | 9 | 1.92E-4 |
|  | MR Egger | 30.15 | 8 | 1.99E-4 |
| Acetoacetate | IVW | 15.88 | 10 | 0.10 |
|  | MR Egger | 15.56 | 9 | 0.08 |
| 3-Hydroxybutyrate | IVW | 20.85 | 25 | 0.70 |
|  | MR Egger | 20.80 | 24 | 0.65 |

**Univariable MR pleiotropy and heterogeneity test for the associations between oily-fish intake and potential mediators**

| **Horizontal pleiotropy test** | | | | |
| --- | --- | --- | --- | --- |
| **Exposure** | **Method** | **Egger_intercept** | **Intercept_se** | **P_intercept_** |
| Uridine | MR Egger | -5.78E-04 | 1.13E-03 | 0.61 |
| Free cholesterol to total lipids ratio in large HDL | MR Egger | 6.76E-03 | 3.05E-03 | 0.03 |
| Total cholesterol in large HDL | MR Egger | 0.01 | 7.83E-03 | 0.20 |
| Acetoacetate | MR Egger | -3.52E-03 | 2.93E-03 | 0.23 |
| 3-Hydroxybutyrate | MR Egger | -2.83E-03 | 2.76E-03 | 0.31 |
| **Heterogeneity test** | | | | |
| **Exposure** | **Method** | **Q statistic** | **Q_df** | **P_heterogeneity_** |
| Uridine | IVW | 38.51 | 47 | 0.81 |
|  | MR Egger | 38.25 | 46 | 0.78 |
| Free cholesterol to total lipids ratio in large HDL | IVW | 114.78 | 60 | 2.68E -05 |
|  | MR Egger | 105.94 | 59 | 1.72E -04 |
| Total cholesterol in large HDL | IVW | 88.83 | 58 | 5.69E-03 |
|  | MR Egger | 86.27 | 57 | 7.41E-03 |
| Acetoacetate | IVW | 86.47 | 60 | 0.01 |
|  | MR Egger | 84.39 | 59 | 0.02 |
| 3-Hydroxybutyrate | IVW | 76.32 | 60 | 0.08 |
|  | MR Egger | 74.98 | 59 | 0.08 |

**Table S10. Mediation effect of each mediator and proportion of each mediation effect.**

| **Cheese intake** | **mediation effects** | **proportion of mediation effects** |
| --- | --- | --- |
| Triglycerides | -0.002 (-0.023 - 0.020) | 0.030 |
| Total cholesterol in large HDL | -0.002 (-0.049 - 0.045) | 0.036 |
| Cholesterol to total lipids ratio in large HDL | -0.001 (-0.033 - 0.031) | 0.026 |
| Free cholesterol to total lipids ratio in large HDL | -0.001 (-0.018 - 0.015) | 0.023 |
| Glycine | 0.001 (-0.035 - 0.037) | 0.022 |
| Phospholipids to total lipids ratio in very large HDL | -0.001 (-0.024 - 0.021) | 0.028 |
| **Non-oily fish intake** | **mediation effects** | **proportion of mediation effects** |
| Uridine | 0.016 (-0.008 - 0.040) | 0.162 |
| Free cholesterol to total lipids ratio in large HDL | 0.011 (-1.269 - 1.290) | 0.112 |
| Total cholesterol in large HDL | 0.010 (-1.316 - 1.335) | 0.103 |
| Acetoacetate | 0.011 (-0.089 - 0.110) | 0.111 |
| 3-Hydroxybutyrate | 0.008 (-0.051 - 0.068) | 0.091 |
| **Oily fish intake** | **mediation effects** | **proportion of mediation effects** |
| Uridine | 0.006 (-0.015- 0.028) | 0.488 |
| Free cholesterol to total lipids ratio in large HDL | -0.002 (-0.020 - 0.017) | 0.318 |

Figure 1 Overview of the MR study design. HDL, high-density lipoprotein; MR, Mendelian randomization.


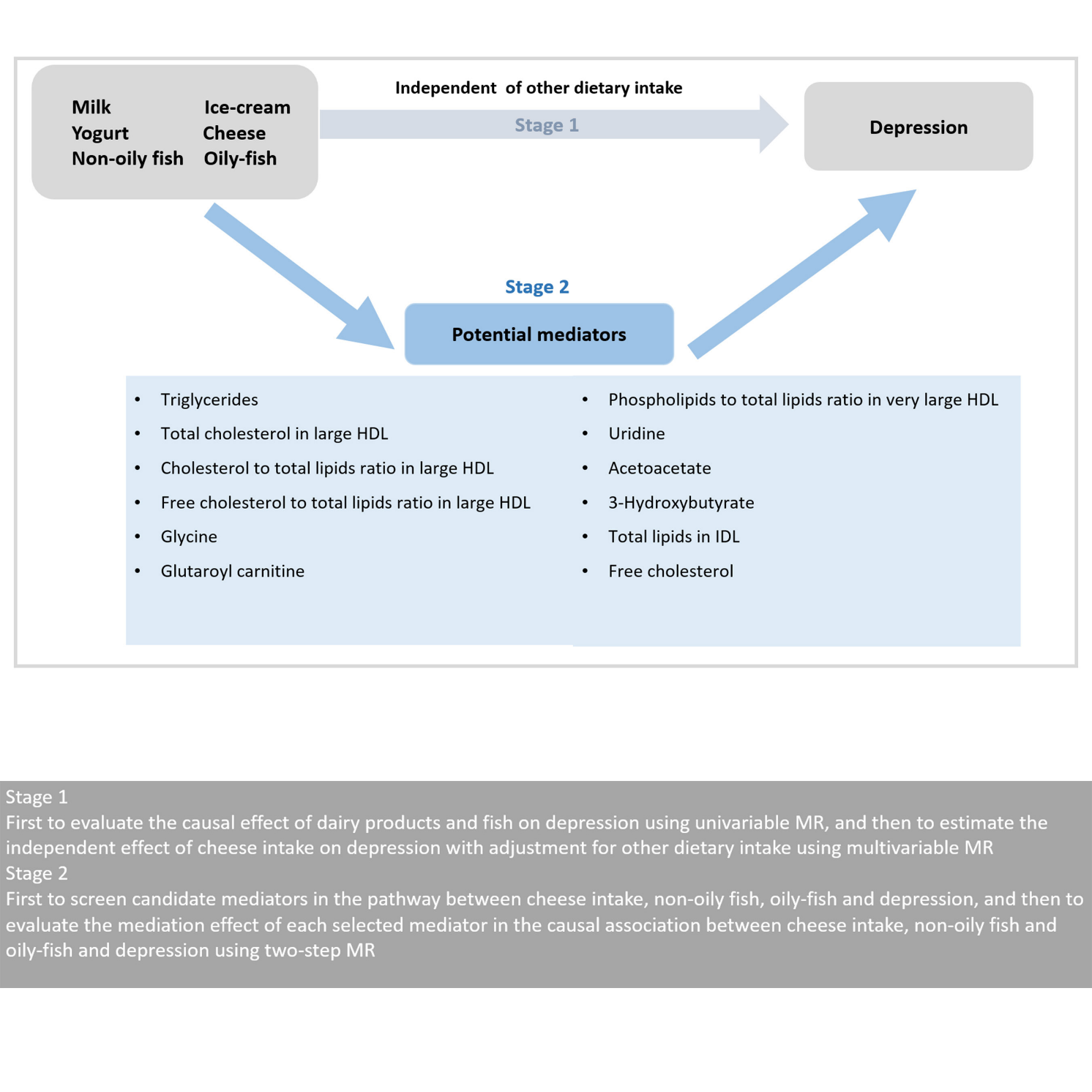


Figure 2 (A) Effect of chesse intake on depression assessed by the primary MR analyses from the GWAS dataset; (B) MR estimates for the causal effects of cheese intake on mediators (left) and the causal effects of mediators on depression (right); (C) MR estimates for the causal effects of non-oily fish on mediators (left) and the causal effects of mediators on depression (right); (D) MR estimates for the causal effects of oily fish on mediators (left) and the causal effects of mediators on depression (right).


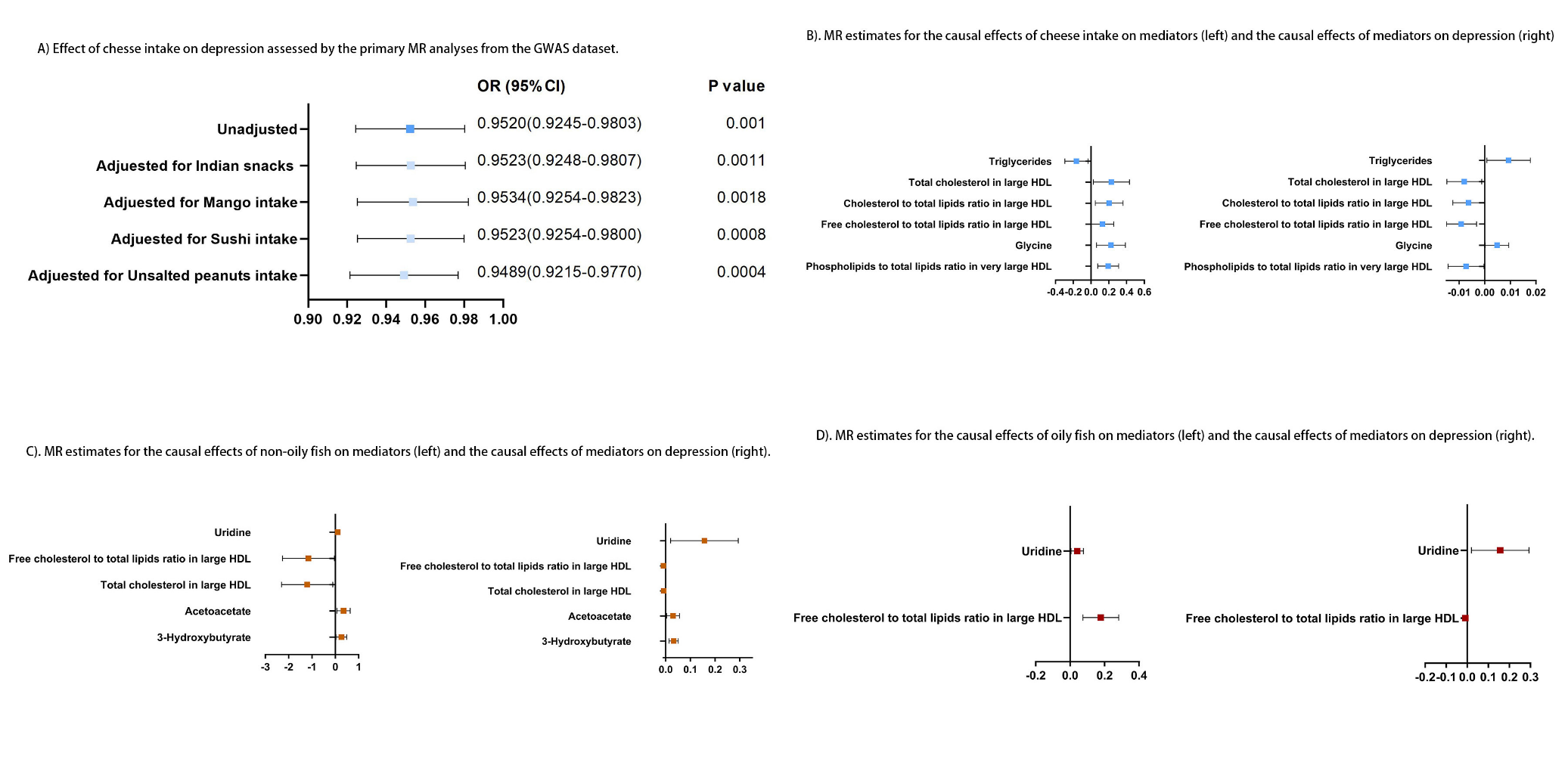


Figure 3 Evidence for selection of mediators in the association between dietary intake and depression. (A) Causal associations between candidate mediators and depression for cheese intake; (B) Casual associations between cheese intake and candidate mediators which were confirmed to causally influence depression; (C) Causal associations between candidate mediators and depression for non-oily fish intake; (D) Casual associations between non-oily fish intake and candidate mediators which were confirmed to causally influence depression; (E) Causal associations between candidate mediators and depression for oily fish intake; (F) Casual associations between oily fish intake and candidate mediators which were confirmed to causally influence depression. ‘Selection’ indicates whether the candidate mediators were selected for subsequent analyses. The IVW method was used for the main analysis. Sensitivity analyses included the MR-Egger and WM shown in the figure, as well as simple mode and weighted mode methods shown in Tables S6 and S8. FDR, false discovery rate; IVW, inverse variance weighted.


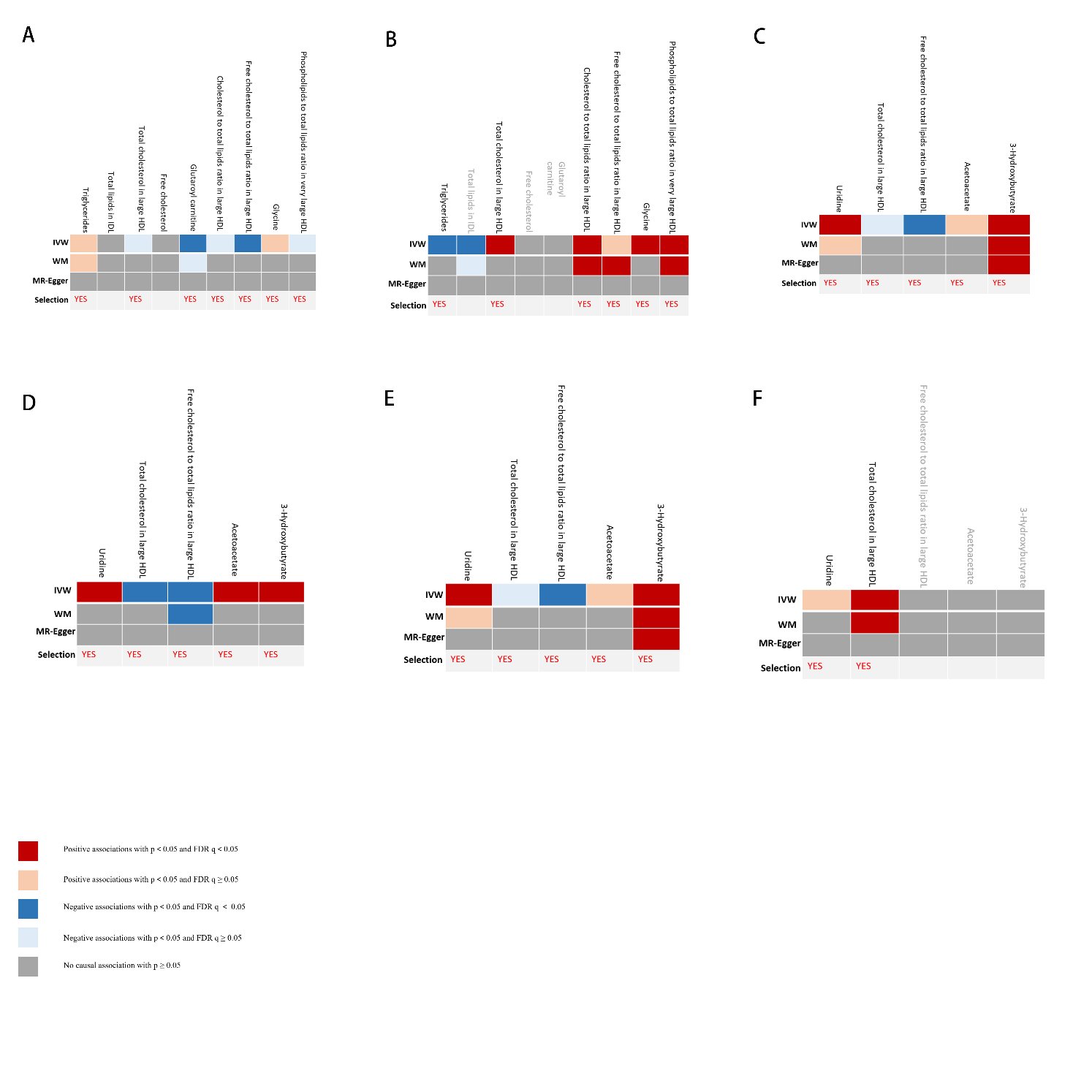


Figure 4 (A) Forest plots of Leave-one-out analyses for causal SNP effect of cheese intake on depression. The error bars indicate the 95% confidence interval (CI). (C) Scatter plots for causal SNP effect of cheese intake on depression. Each black point representing each SNP on the exposure (horizontal-axis) and on the outcome (vertical-axis) is plotted with error bars corresponding to each standard error (SE). The slope of each line corresponds to the combined estimate using each method of the inverse variance weighted (light blue line), the MR-Egger (blue line), the simple mode (light green line), the weighted median (green line), and the weighted mode (pink line). SNP: single nucleotide polymorphism, MR, Mendelian randomization.


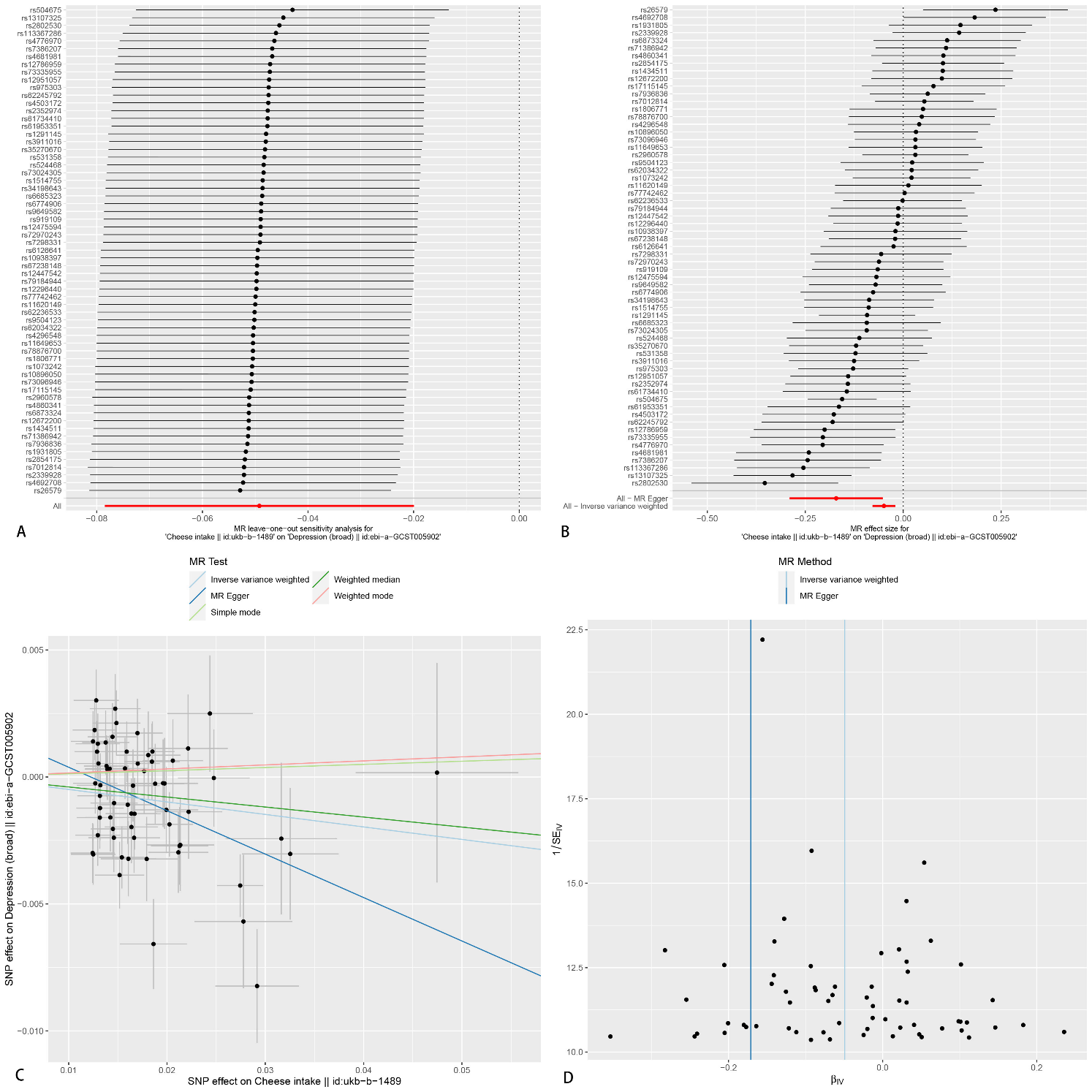


Figure 5 (A) Two-step MR analysis framework; (B) DAG for the proposed causal interactions of metabolites in the pathway between dietary intake and depression. The black arrows represent causal pathways from dietary intake, through mediators identified in this MR study, to depression. The grey arrows represent other plausible pathways linking dietary intake to depression that we did not investigate in this study. DAG, directed acyclic diagram; MR, Mendelian randomization.


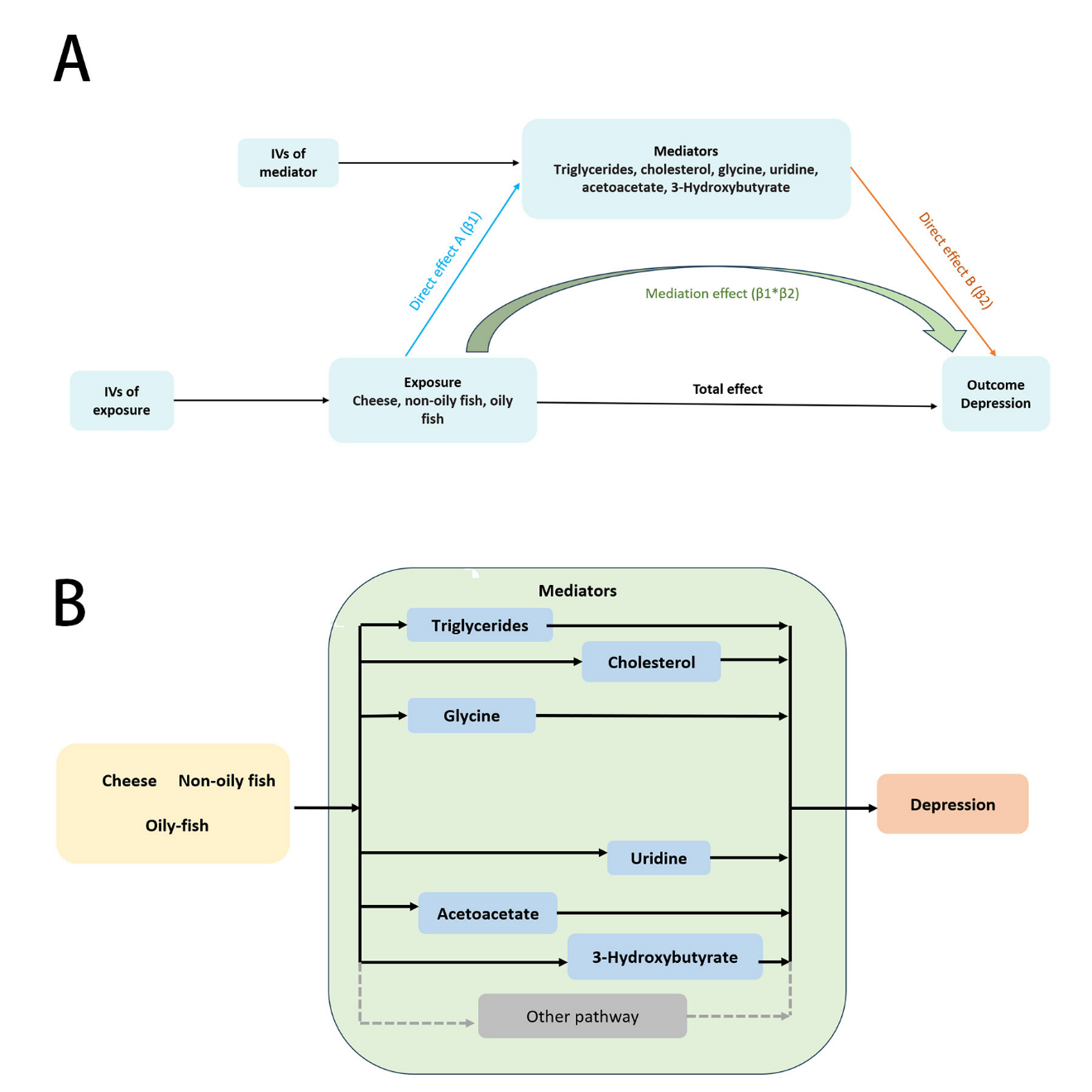


Figure 6 (A) The enrichment analysis of pathways of cheese intake for the treatment of depression. (B) The enrichment analysis of pathways of non-oily fish intake for the treatment of depression. (C) The enrichment analysis of pathways of oily fish intake for the treatment of depression. (D) Eight key metabolic pathways of of non-oily fish intake for the treatment of depression


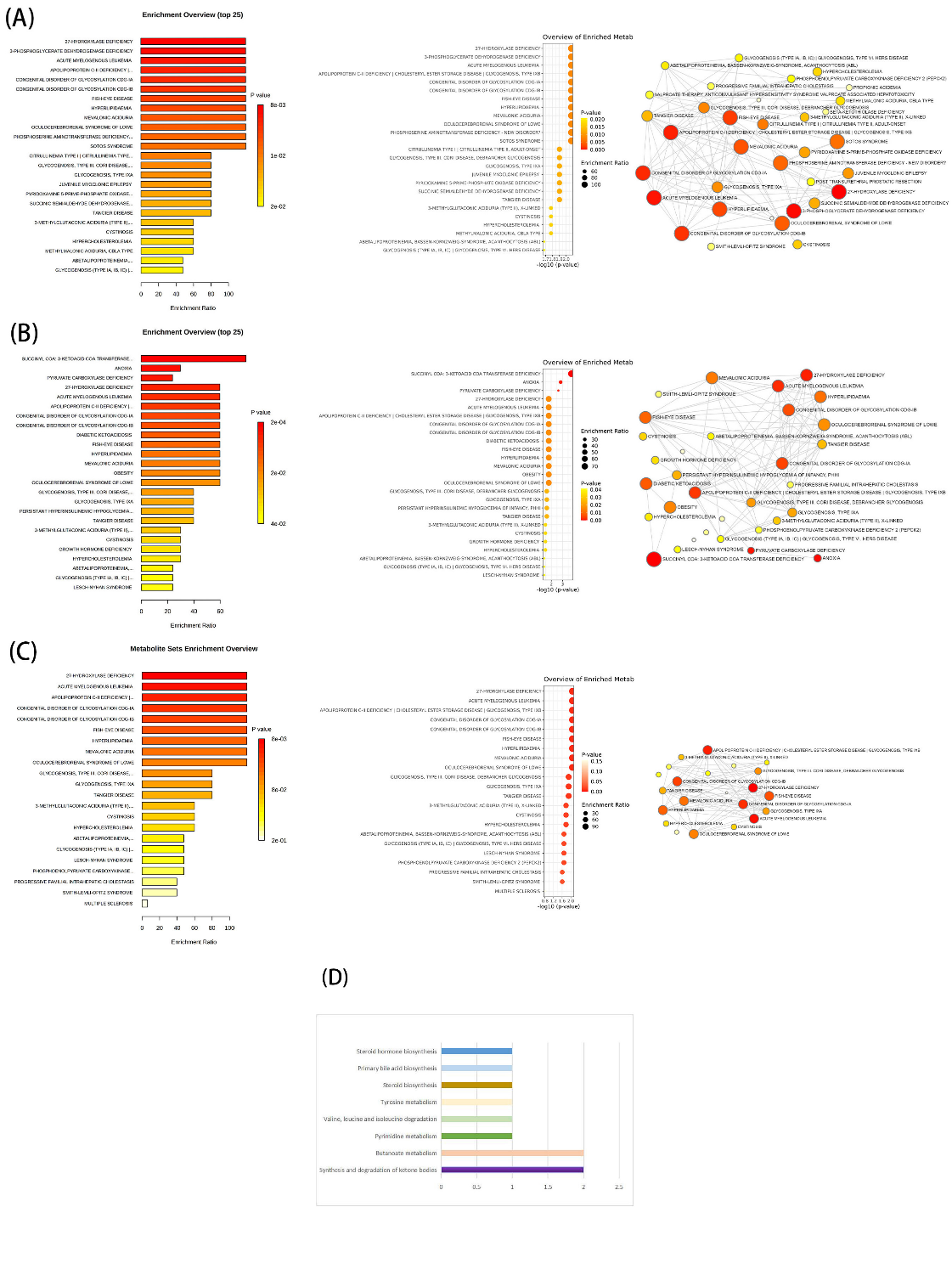


Figure 7 Molecular docking. Binding mode of glycine with Glutamate Receptor 2.


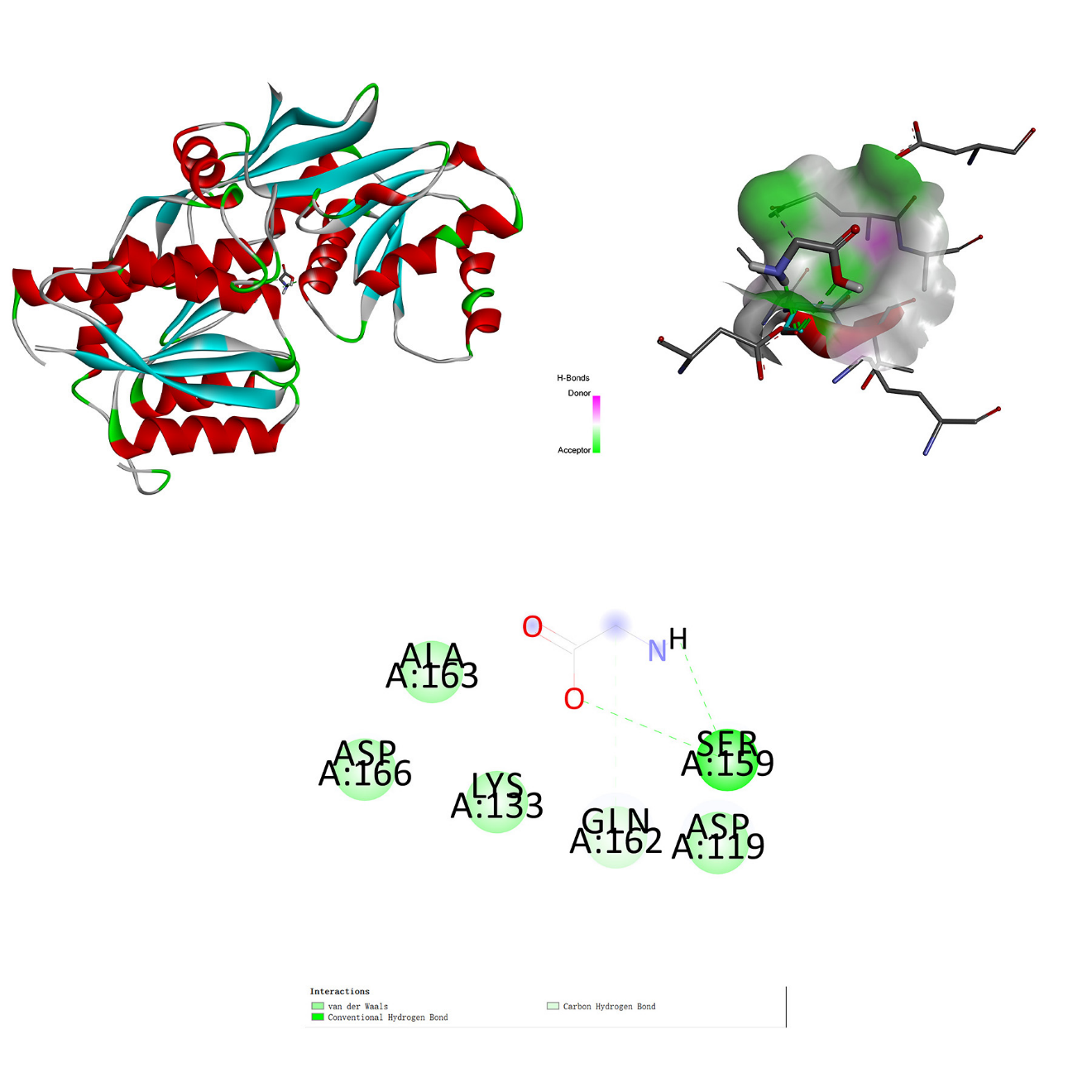


**
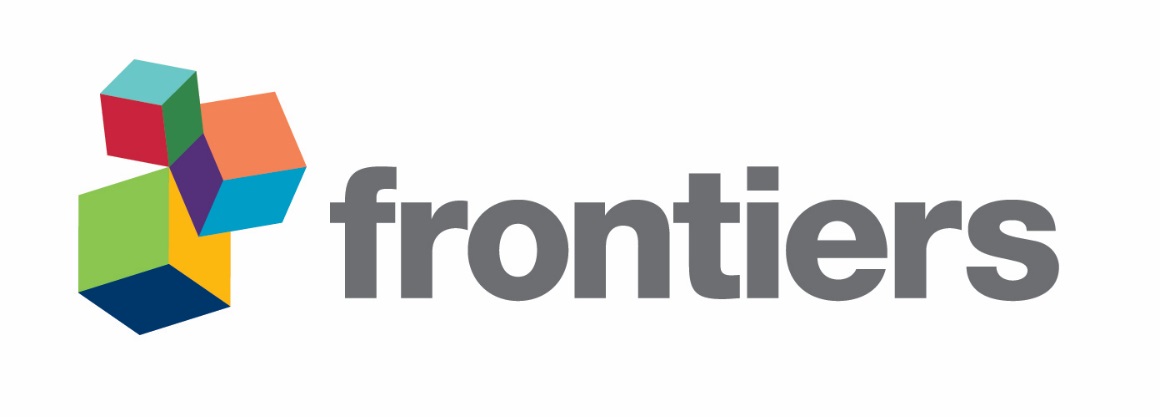
**
